# Synthesis, aromatization and cavitates of an oxanorbornene-fused dibenzo[*de*,*qr*]tetracene nanobox†

**DOI:** 10.1039/d1sc06553j

**Published:** 2022-01-13

**Authors:** Han Chen, Zeming Xia, Qian Miao

**Affiliations:** Department of Chemistry, The Chinese University of Hong Kong Shatin New Territories Hong Kong China miaoqian@cuhk.edu.hk

## Abstract

Oxanorbornene-fused double-stranded macrocycles, represented by kohnkene, are not only synthetic precursors toward short segments of zigzag carbon nanotubes but also typical cavitands processing an intrinsic cavity. However, their capability to bind guest molecules in solution remained unexplored. Herein we report a new member of oxanorbornene-fused double-stranded macrocycles, which is named a nanobox herein because of its shape. Reductive aromatization of this oxanorbornene-fused nanobox leads to observation of a new zigzag carbon nanobelt by high resolution mass spectroscopy. The fluorescence titration and NMR experiments indicate that this nanobox encapsulates C_70_ in solution with a binding constant of (3.2 ± 0.1) × 10^6^ M^−1^ in toluene and a high selectivity against C_60_ and its derivatives. As found from the X-ray crystallographic analysis, this nanobox changes the shape of its cross-section from a rhombus to nearly a square upon accommodating C_60_.

## Abstract 2

A new oxanorbornene-fused nanobox encapsulated C_70_ selectively in solution with a binding constant of (3.2 ± 0.1) × 10^6^ M^−1^. Reductive aromatization of this nanobox led to observation of a new zigzag carbon nanobelt by mass spectroscopy.

## Graphical abstract

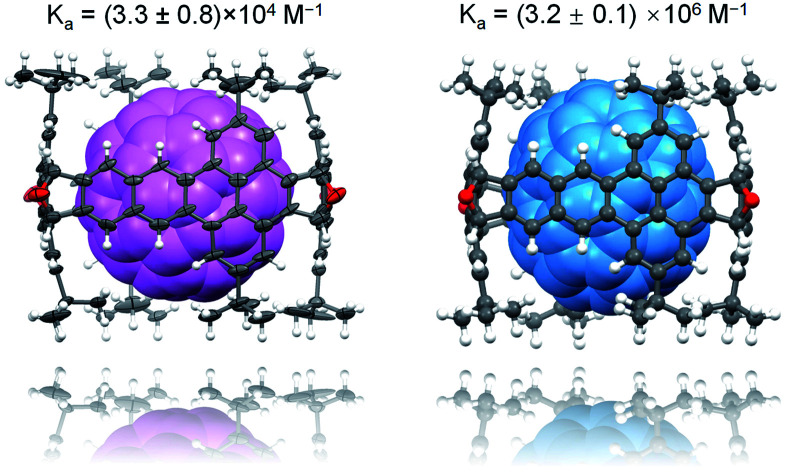

## Introduction

**Fig. 1:**
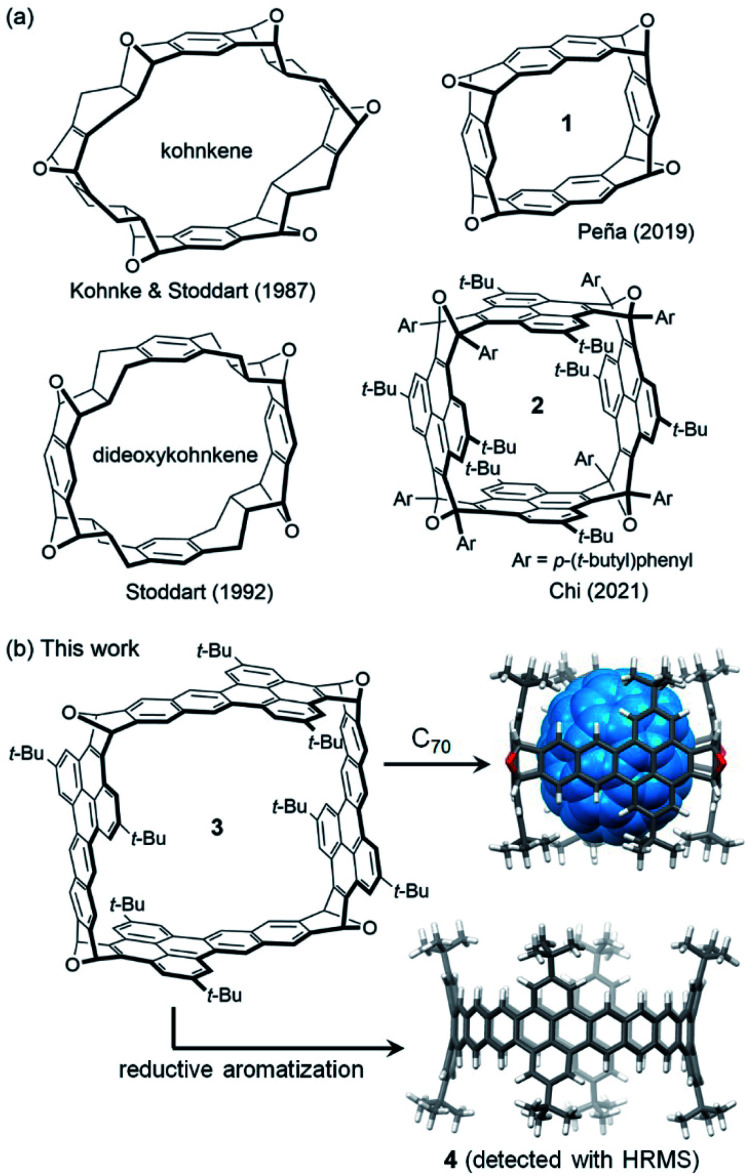
(a) Structures of known oxanorbornene-fused double-stranded macrocycles; (b) structures of nanobox 3 and the energy-minimized models of carbon nanobelt 4 and C_70_⊂3 as calculated at the B3LYP-D3 level of DFT with the 6-31g(d) basis set.

Oxanorbornene-fused double-stranded macrocycles, represented by kohnkene (Fig. 1a), have received considerable attention not only because they are synthetic precursors toward short segments of zigzag carbon nanotubes^1–6^ but also because they are typical cavitands processing well defined cavities as a result of the intrinsic curvature of the oxanorbornene moieties.^7^ Kohnkene was synthesized by Kohnke, Stoddart and co-workers in 1987 during the targeted synthesis of [12]cyclacene,^8,9^ and named after its creator. Partial deoxygenation of kohnkene with low valent titanium gave dideoxykohnkene^10^ (Fig. 1a), which has a cavity shaped like a Celtic cross. The recently revived interest in the synthesis of carbon nanobelts,^11–13^ particularly, the efforts to synthesize zigzag carbon nanobelts^14–16^ by Diels–Alder reactions^17–20^ gave rise to new members of oxanorbornene-fused double-stranded macrocycles,^21^ such as 1^17^ and 2 ^20^ (Fig. 1a). Because these double-stranded macrocycles are shaped like boxes, they are named oxanorbornene-fused nanoboxes herein. Similar to kohnkene and dideoxykohnkene, these nanoboxes all have a well-defined cavity although they lack hydrogen atoms pointing inward the cavity. However, their potential to function as molecular containers is largely unexplored. It is found that kohnkene has a cavity too small to accommodate any molecular guest, while dideoxykohnkene can only accommodate a molecule of water in its cavity in the crystal state. Some oxanorbornene-fused nanoboxes (*e.g.*1) are found to have their cavities occupied by co-crystallized solvent molecules,^17,21^ while the others (*e.g.*2) are found to have empty cavities in the crystals because access to the cavity is blocked by bulky substituting groups.^18,20^ On the other hand, none of these oxanorbornene-fused macrocycles have demonstrated the capability of accommodating guest molecules in solution.

Herein we report a new oxanorbornene-fused nanobox (3 in Fig. 1b), which contains four dibenzo[*de*,*qr*]tetracene subunits. Density functional theory (DFT) calculations indicate that the cavity of 3 has a cross-section shaped like a square and can accommodate a fullerene, such as C_70_ (Fig. 1b). As detailed below, reductive aromatization of 3 led to observation of the corresponding zigzag carbon nanobelt 4 (Fig. 1b) by high resolution mass spectroscopy, and the capability of 3 to bind a fullerene (C_60_ or C_70_) in both solution and crystal states was demonstrated using different techniques.

## Results and discussion

### Synthesis and structural analysis

**Scheme 1:**
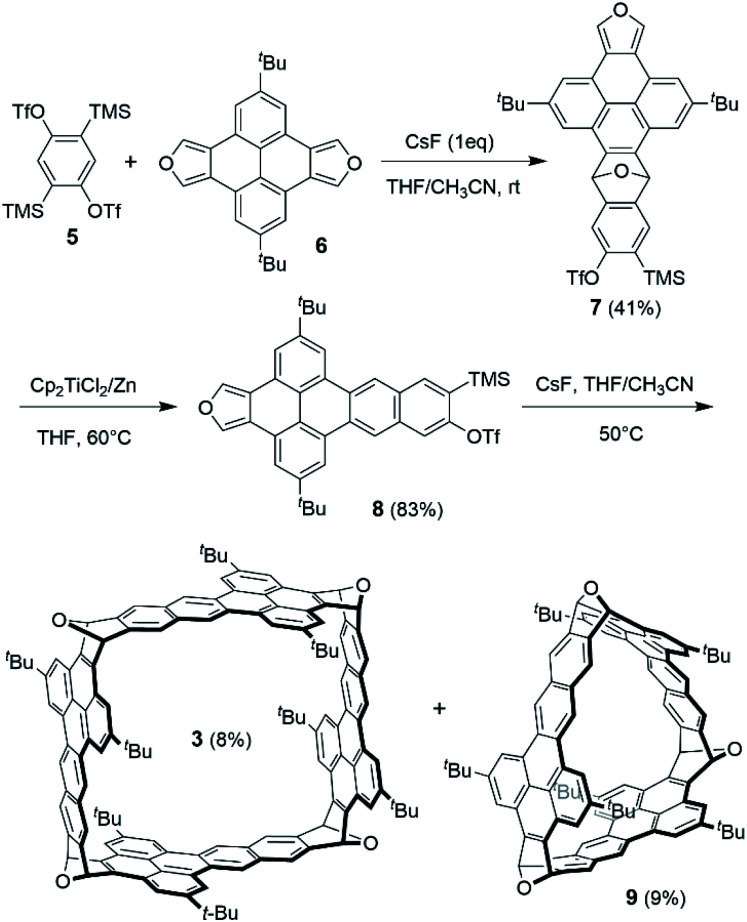
Synthesis of 3.

Scheme 1 shows the synthesis of 3 starting from bistriflate 5^22^ and pyrenodifuran 6,^18^ which were synthesized following the reported procedures. Treatment of 5 with one equivalent of CsF resulted in the corresponding benzyne *in situ*, which reacted with one equivalent of 6 to afford the Diels–Alder adduct (7) in a yield of 41%. Reductive aromatization of 7 with TiCp_2_Cl_2_/Zn^23^ gave the deoxygenated product (8) in a yield of 83%. In contrast, the attempts to deoxygenate 8 under other conditions including NaI/trimethylsilyl iodide (TMSI)^17,18^ and NH_4_ReO_4_/P(OPh)_3_ ^24^ led to decomposition of 7 or a very low yield of 8. Having a diene moiety (in the furan ring) on one end and a potential benzyne (to be formed by desilylation and elimination of triflate) as the dienophile on the other end, 8 was used as a bifunctional building block to construct the nanobox through Diels–Alder reactions. The reaction of 8 with an excess of CsF (5 equivalents) in a dilute solution in THF and acetonitrile at 50 °C gave the cyclic tetramer (3) in a yield of 8% together with the cyclic trimer (9) in a yield of 9%. The yields of 3 and 9 are higher than those of the reported oxanorbornene-fused nanoboxes from a bisbenzyne precursor and a bisfuran in a two-step manner^18^ (*e.g.* 4% for compound 1^17^ and 2% for compound 2^20^). The pale-yellow solids of 3 and 9 both form colorless solutions in CH_2_Cl_2_, which both exhibit blue luminescence upon irradiation with UV light. As shown in Fig. S2 in the ESI,† the absorption and photoluminescence spectra of 3 are very similar to those of 9, respectively, but have higher intensity, in agreement with the fact that 3 has more dibenzo[*de*, *qr*]tetracene subunits than 9. The ^1^H NMR of 3 in the downfield region shows five singlets (Fig. S31†), which are assigned to the corresponding protons on the basis of the ROESY 2D NMR (Fig. S32†). The ^1^H NMR of 9 in the downfield region (Fig. S29†), slightly different from that of 3, shows three singlets and two doublets due to observation of the coupling between two meta protons on the same benzene ring.

**Fig. 2:**
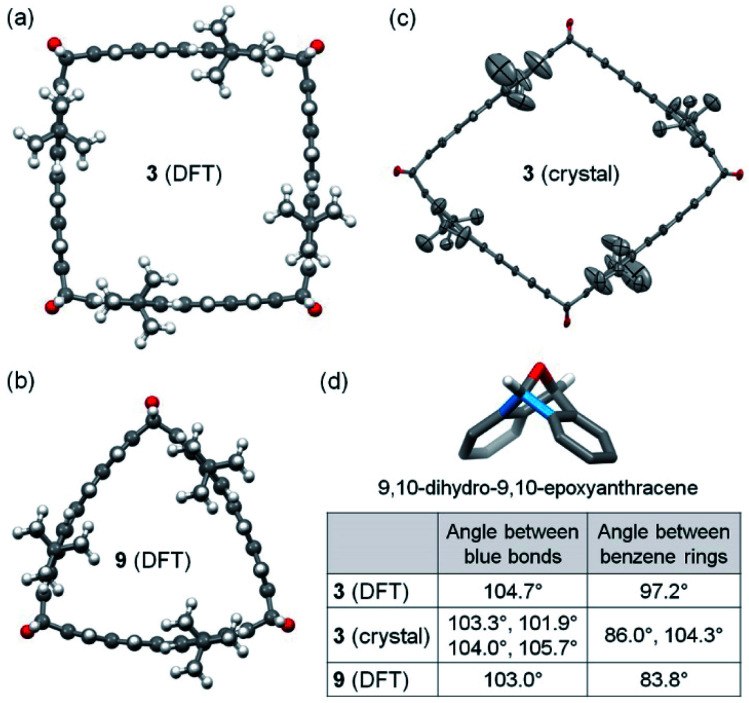
(a) Energy-minimized model of 3 calculated at the B3LYP-D3 level of DFT; (b) energy-minimized model of 9; (c) structure of 3 in the crystal (carbon and oxygen atoms are shown as ellipsoids at the 50% probability level, hydrogen atoms are removed for clarity); (d) summary of the bond angle and the dihedral angle between benzene rings in the 9,10-dihydro-9,10-epoxyanthracene moiety in 3 and 9.

As revealed by the calculations at the B3LYP-D3 level of DFT with the 6-31g(d) basis set, 3 is of *C*_4h_ symmetry and has a slightly bent square cross-section with essentially flat π-planes of dibenzo[*de*,*qr*]tetracene (Fig. 2a), while 9 is of *C*_3h_ symmetry and its cross-section is shaped like a Reuleaux triangle with bent π-planes of dibenzo[*de*,*qr*]tetracene (Fig. 2b). The 9,10-dihydro-9,10-epoxyanthracene moiety at each corner of 3 exhibits the same bond angle of 104.7° between the two blue C–C bonds as shown in Fig. 2d, while that of 9 exhibits a slightly smaller bond angle of 103.0° at each corner. On the basis of the hypothetical homodesmotic reactions shown in Fig. S17 in the ESI,† the strain energy of 3 and 9 is estimated as 7 kcal mol^−1^ and 16 kcal mol^−1^, respectively. Although 3 is less strained than 9, 3 was obtained in a slightly lower yield presumably because the formation of 3 requires one more Diels–Alder cycloaddition. Single crystals of 3 were obtained by slow diffusion of isopropanol vapor into its solution in CH_2_Cl_2_ and 1,2-dichloroethane. X-ray crystallography revealed a triclinic unit cell containing one molecule of 3 and co-crystallized 1,2-dichloroethane and CH_2_Cl_2_.^25^ It is found that 3 in the crystal has a roughly rhombic cross-section (Fig. 2c) with four different bond angles (101.9–105.7°) in the 9,10-dihydro-9,10-epoxyanthracene moieties as shown in Fig. 2d. The different bond angles (Fig. 2d) and the different dihedral angles between benzene rings in the 9,10-dihydro-9,10-epoxyanthracene moieties in 3 (both DFT-calculated model and crystal structure) and 9 indicate that the oxanorbornene units in these double-stranded macrocycles are not completely rigid but flexible to some degree.

### Reductive aromatization of the oxanorbornene-fused nanoboxes

**Fig. 3:**
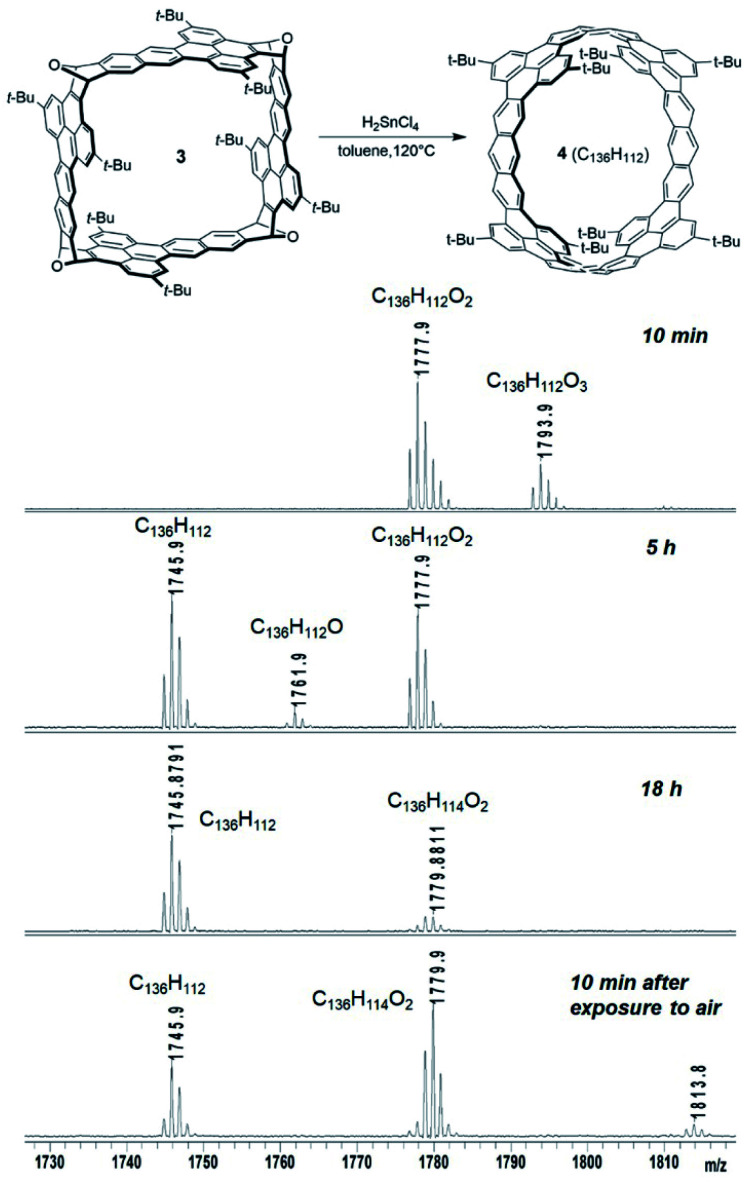
Reductive aromatization of 3 as monitored by mass spectroscopy.

Because reductive aromatization of 3 and 9 can in principle result in the corresponding zigzag carbon nanobelts, the deoxygenation reactions of 3 and 9 were tested under different conditions including H_2_SnCl_4_, TiCpCl_2_/Zn,^23^ NaI/TMSI,^26^ TiCl_4_/LiAlH_4_,^27^ and NH_4_ReO_4_/P(OPh)_3_.^24^ Among these conditions, only treatment with H_2_SnCl_4_ ^28^ (freshly prepared from anhydrous SnCl_2_ and concentrated HCl) at 120 °C under an atmosphere of N_2_ was able to convert 3 to the corresponding carbon nanobelt (4), which was detected by MALDI-TOF mass spectroscopy from the crude product. As shown in Fig. 3, when 3 was treated with H_2_SnCl_4_ in toluene at 120 °C for 10 minutes, the mass spectrum from the reaction mixture indicated the formation of partially deoxygenated products C_136_H_112_O_3_ and C_136_H_112_O_2_. When the reaction time was prolonged, 4 (C_136_H_112_) gradually became the major product. The observed molecular ion peak (*m*/*z* of 1745.8791) and isotope patterns (Fig. 3 and S24†) are in good agreement with the molecular formula of C_136_H_112_ (*m*/*z* of 1745.8792). When the crude product was cooled to room temperature and exposed to air, the mass spectrum exhibited a new peak of *m*/*z* = 1779.8811 for C_136_H_114_O_2_, which likely resulted from photo-induced oxygenation of 4 by molecular oxygen in air followed by protonation. This peak increased and the peak of 4 decreased quickly as exposure to air was prolonged (Fig. 3). This indicates low stability of 4 toward oxidation by air. Temperature was found important to the reduction of 3 with H_2_SnCl_4_. When treated with H_2_SnCl_4_ at room temperature for 5 hours, 3 was almost completely recovered. When treated with H_2_SnCl_4_ at 80 °C for 5 hours, 3 was partially recovered and a partially deoxygenated product (C_136_H_112_O_2_) was observed by mass spectroscopy. In contrast, other conditions either led to only partial deoxygenation or gave over-reduced products that exhibited molecular ion peaks in the mass spectra in agreement with hydrogenated carbon nanobelts. Unfortunately, our attempts to isolate 4 were not successful. When the reaction mixture was extracted and concentrated, the molecular ion peak for 4 disappeared and the product became less soluble, likely due to oligomerization and oxidation. The nanobelt 4 is less stable than the successfully synthesized zigzag carbon nanobelts^19,20^ likely because 4 has fewer aromatic sextets.

### Cavitates of the nanobox with fullerenes

**Fig. 4:**
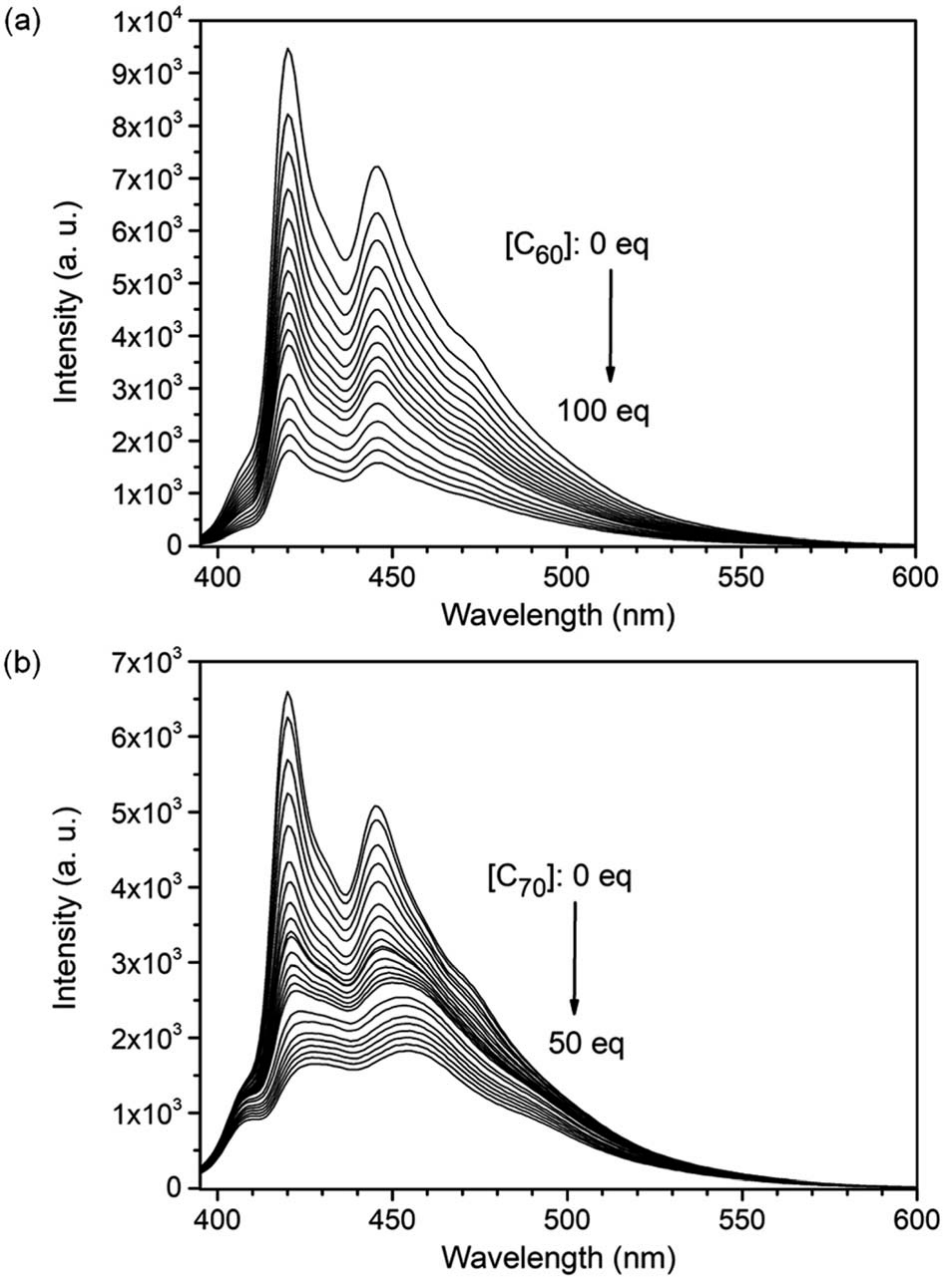
Fluorescence spectra of (a) 3 (5.0 × 10^−7^ mol L^−1^) in toluene titrated with C_60_ (from 0 to 5.0 × 10^−5^ mol L^−1^) and (b) 3 (1.0 × 10^−7^ mol L^−1^) in toluene titrated with C_70_ (from 0 to 5.0 × 10^−6^ mol L^−1^) at room temperature.

Host–guest chemistry of the nanobox 3 in solution with different fullerenes including C_60_, C_70_, [6,6]-phenyl-C_61_-butyric acid methyl ester (PCBM) and indene-C_60_ bisadduct (ICBA) was studied using different techniques. From a solution containing a 1 : 1 mixture of 3 and C_60_ or a derivative of C_60_ in toluene, the high resolution MALDI-TOF mass spectra (Fig. S20–S22 in the ESI†) revealed both free 3 and the corresponding cavitate: C_60_⊂3 (*m*/*z*: 2530.8703), PCBM⊂3 (*m*/*z*: 2720.9606), or ICBA⊂3 (*m*/*z*: 2762.9836). In contrast, from the solution of 3 and C_70_ (1 : 1) in toluene, only the molecular ion peak for C_70_⊂3 (*m*/*z*: 2650.8664) was observed in the mass spectrum (Fig. S23†). This suggests that 3 binds C_70_ more strongly than C_60_. Upon gradual addition of a fullerene to the solution of 3, the intensity of the blue fluorescence of 3 decreased dramatically as shown in Fig. 4, S9 and S11.† The Job's plots with an unchanged total concentration of 3 and fullerene in toluene showed a maximum fluorescence change when the ratio of 3:fullerene reached 1 : 1 as shown in Fig. S3–S6.† On the basis of the 1 : 1 stoichiometry, the binding constant (*K*_a_) of 3 for C_60_ is determined as (3.3 ± 0.8) × 10^4^ M^−1^ at room temperature by fitting the data from three independent fluorescence titration experiments using the equation: *F*/*F*_0_ = (1 + (*k*_f_/*k*_s_)*K*_a_[C_60_])/(1 + *K*_a_[C_60_]),^29,30^ where *F* and *F*_0_ are the fluorescence intensity of nanobelts with and without addition of C_60_, respectively; *k*_f_ and *k*_s_ are proportionality constants for the complex and nanobox 3, respectively; and *K*_a_ is the binding constant of nanobox 3 for C_60_. Using the same method, the binding constants of 3 for C_60_ derivatives, PCBM and ICBA, are determined as (3.3 ± 0.9) × 10^4^ M^−1^ and (3.1 ± 0.7) × 10^4^ M^−1^, respectively, which are essentially the same as that of C_60_⊂3 likely as a result of arranging the substituting groups outside the cavity of 3. The binding constant of 3 for C_70_ at room temperature is determined using the same method as (3.2 ± 0.1) × 10^6^ M^−1^, which is larger than that of C_60_⊂3 by two orders of magnitude. The binding constant of 3 for C_70_ in toluene is larger than the reported values of [10]cycloparaphenylene ((8.4 ± 0.3) × 10^4^ M^−1^, measured from UV-vis titration),^31^ [11]cycloparaphenylene ((1.5 ± 0.1) × 10^5^ M^−1^, measured from UV-vis titration),^31^ and [4]cyclo(2,11-hexa-*peri*-hexabenzocoronene) (1.07 × 10^6^ M^−1^, measured from fluorescence quenching)^32^ but lower than that of porphyrin nanohoops (2 × 10^7^ M^−1^, measured from UV-vis titration)^33^ in the same solvent. Moreover, 3 exhibits a higher selectivity between two fullerenes (C_70_/C_60_ = 97) than [10]cycloparaphenylene (C_60_/C_70_ = 33),^30,31^ the porphyrin nanohoop (C_60_/C_70_ = 15),^33^ and (12,8)-[4]cyclo-2,8-anthanthrenylene (C_70_/C_60_ = 1.3 in *o*-dichlorobenzene)^34^ as well as the self-assembled capsule (C_70_/C_60_ = 21 in C_2_H_2_Cl_4_ as measured from UV-vis titration or C_70_/C_60_ = 4.2 in C_2_H_2_Cl_4_ as measured by isothermal titration calorimetry).^35^

**Fig. 5:**
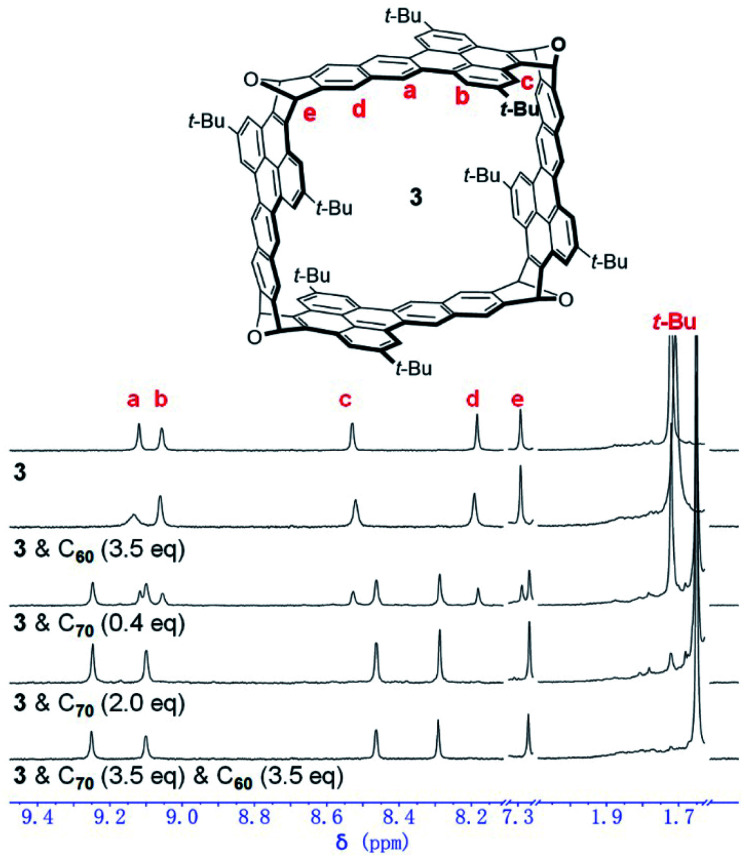
^1^H NMR spectrum of 3 in *o*-C_6_D_4_Cl_2_ in comparison to those of 3 with C_60_ or C_70_ in *o*-C_6_D_4_Cl_2_ (400 MHz, 298 K).

In order to study the encapsulation of C_60_ and C_70_ by 3 with NMR spectroscopy, *o*-C_6_D_4_Cl_2_, a better solvent for fullerenes, was used. Addition of excessive C_60_ (3.5 eq.) into the solution of 3 in *o*-C_6_D_4_Cl_2_ led to a broadened and slightly down-field shifted peak for H-a as shown in Fig. 5. In contrast, addition of 0.4 equivalent of C_70_ into the solution of 3 resulted in two apparently different sets of peaks, which are attributed to the free host (3) and the complex (C_70_⊂3), respectively. In the presence of excessive C_70_ (2.0 eq.), the peaks for the free host disappeared and only the peaks for C_70_⊂3 were observed. The same ^1^H NMR spectrum (Fig. 5) was observed when 3.5 eq. of C_70_ was added into a solution that already contained 3 and 3.5 equivalent of C_60_ in *o*-C_6_D_4_Cl_2_. These results indicate that 3 binds C_60_ weakly in *o*-C_6_D_4_Cl_2_ with a fast exchange at the NMR time scale but binds C_70_ strongly with a slow exchange under the same conditions. In agreement with the NMR experiments, from the solution containing a 1 : 1 : 1 mixture of 3, C_60_ and C_70_ in *o*-C_6_H_4_Cl_2_, the high-resolution mass spectrum revealed the complex of C_70_⊂3 only. These results indicate selective encapsulation of C_70_ by 3 in the presence of C_60_. From the NMR titration experiments (Fig. S36 in the ESI†), the binding constant of 3 for C_70_ in *o*-C_6_H_4_Cl_2_ at room temperature is determined as 2.5 × 10^4^ M^−1^, which is smaller than the binding constant in toluene presumably because C_70_ and 3 have a higher degree of solvation in *o*-C_6_H_4_Cl_2_ than in toluene.

**Fig. 6:**
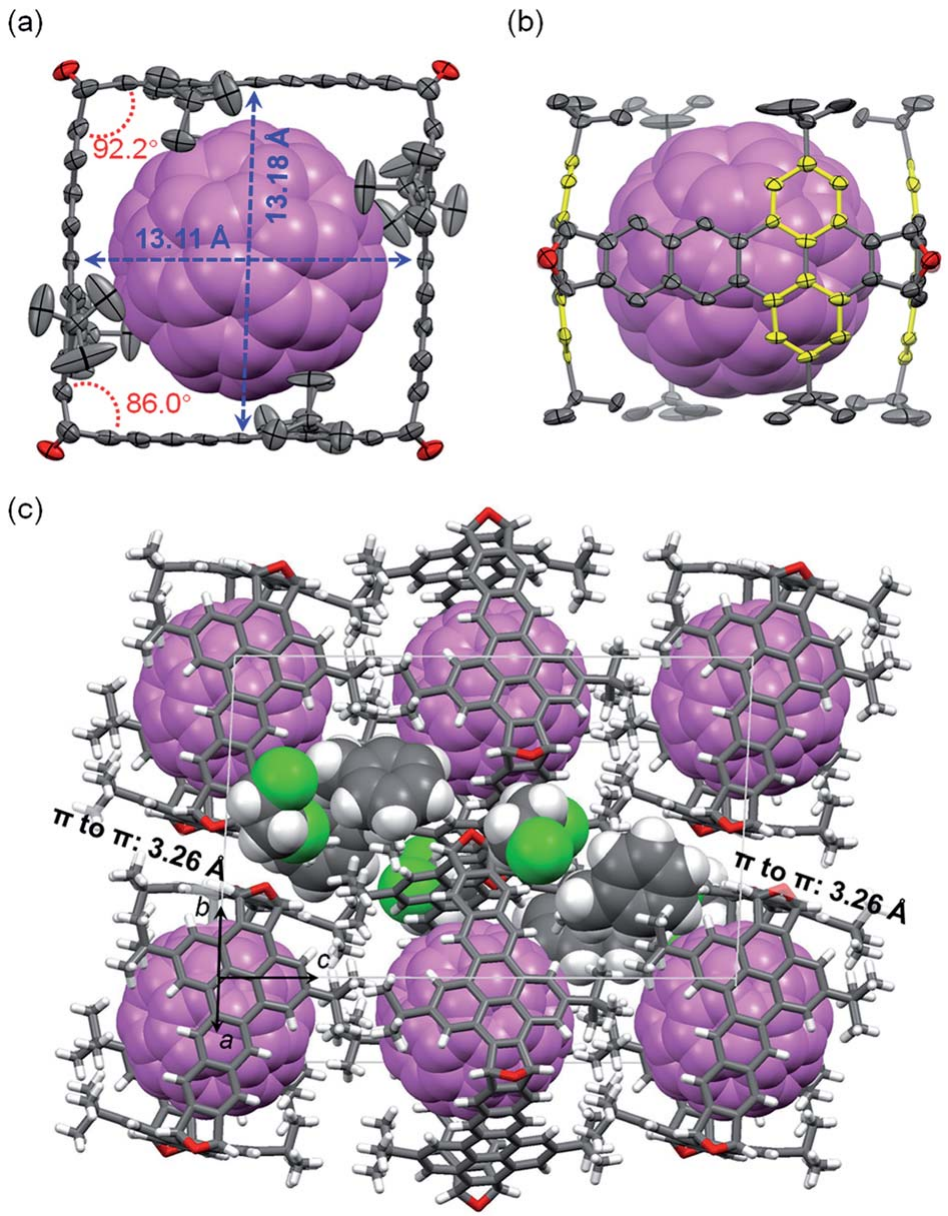
Crystal structure of C_60_⊂3·2(C_2_H_4_Cl_2_)·2(C_6_H_5_CH_3_): (a) top view of C_60_⊂3; (b) side view of C_60_⊂3; (c) molecular packing in a unit cell. C_60_ is shown in violet with a space-filling model; in panels a and b, carbon and oxygen atoms in 3 are shown as ellipsoids at the 50% probability level, and hydrogen atoms are removed for clarity; in panel c, molecules of 3 are shown with stick models, and co-crystallized solvent molecules are shown with space-filling models.

The structure of C_60_⊂3 was unambiguously determined by X-ray crystallographic analysis of the single crystals of C_60_⊂3·2(C_2_H_4_Cl_2_)·2(C_6_H_5_CH_3_),^25^ which were grown by slow diffusion of isopropanol vapor into the solution of C_60_ and 3 in *o*-dichloroethane (C_2_H_4_Cl_2_) and toluene (C_6_H_5_CH_3_). However, our attempts to grow single crystals of C_70_⊂3 suitable for X-ray diffraction were not successful. As shown in Fig. 6a, the cross section of 3 in the crystal of C_60_⊂3 is close to a square, which has a side length of about 13.1 Å as measured from the distance between the center carbon atoms of dibenzo[*de*,*qr*]tetracene on the opposite sides. Sixteen short intermolecular C-to-C contacts in the range of 3.11–3.36 Å are observed between 3 and C_60_. The cross-section of 3 in C_60_⊂3 has inner angles of 92.2° and 86.0° as measured from the dihedral angle between the two benzene rings in each 9,10-dihydro-9,10-epoxyanthracene moiety (see Fig. 2d). Another finding from comparing the structures of 3 in the crystals with and without C_60_ is that the yellow benzene rings in 3 bend inward to form concave–convex π–π interactions with C_60_ as shown in Fig. 6b. As a result, the two yellow benzene rings in the same dibenzo[*de*,*qr*]tetracene unit form a dihedral angle of 13.7°. Further detailed analysis on the Hirshfeld surface^36^ of C_60_ in the complex shows short contacts (CH–π interactions) between the *t*-butyl group of 3 and C_60_ (Fig. S1 in the ESI†). Fig. 6c shows the molecular packing in a unit cell of C_60_⊂3·2(C_2_H_4_Cl_2_)·2(C_6_H_5_CH_3_), where two adjacent molecules of 3 exhibit face-to-face π-stacking with a distance of 3.26 Å between π-planes (defined by the tetracene moiety). In contrast, no π–π interactions are observed between molecules of C_60_, which are in fact separated by the *t*-butyl groups of 3. The π–π stacking between molecules of 3 and the relatively high HOMO energy level of 3 (−5.18 eV as calculated at the B3LYP/6-31-g(d) level of DFT) suggest that the crystals of C_60_⊂ 3 can, in principle, function as hole-transporting organic semiconductors for application in phototransistors or photodetectors^37^ on the basis of photo-induced electron transfer^38^ from 3 to C_60_. Unfortunately, our preliminary efforts to drop-cast or dip-coat a solution of C_60_⊂3 onto a substrate failed to give films suitable for device fabrication.

## Conclusions

In summary, the above study has put forth a new oxanorbornene-fused nanobox (3), which contains four dibenzo[*de*, *qr*]tetracene subunits. It was synthesized through one-pot iterative Diels–Alder reactions, which also gave a Reuleaux triangle-shaped double-stranded macrocycle (9). Reductive aromatization of 3 with H_2_SnCl_4_ led to observation of the corresponding zigzag carbon nanobelt by high resolution mass spectroscopy. The host-guest chemistry of 3 with different fullerenes was studied in both solution and crystal states using different techniques. The fluorescence titration experiments indicate that 3 encapsulates C_70_ in toluene with a binding constant of (3.2 ± 0.1) × 10^6^ M^−1^ and a high selectivity against C_60_ and its derivatives. The NMR titration experiments indicate that 3 encapsulates C_70_ with a slow exchange at the NMR time scale and a binding constant of 2.5 × 10^4^ M^−1^ in *o*-dichlorobenzene. The X-ray crystallographic analysis shows that 3 changes the shape of its cross-section from a rhombus to nearly a square upon accommodating C_60_. On the basis of the above results, 3 is the first member of oxanorbornene-fused double-stranded macrocycles demonstrating the capability to accommodate molecular guests in solution. This aromatic nanobox may find potential application for crystallization of fullerene derivatives, which is still a challenge in fullerene chemistry due to the limited solubility of fullerenes and the geometrical similarities of the carbon spheroids.^39–41^

## Data availability

All the data are provided in ESI.†

## Author contributions

H. Chen and Q. Miao conceived the project, and Q. Miao directed the project. H. Chen performed most of the experiments and calculations, and Z. Xia contributed to the fluorescence tritration experiments and data analysis. Q. Miao and H. Chen wrote the manuscript, and all authors checked the manuscript.

## Conflicts of interest

There are no conflicts of interest to declare.

## Supplementary Material

SC-013-D1SC06553J-s001

SC-013-D1SC06553J-s002
