# Supplementary material for: Synthesis, aromatization and cavitates of an oxanorbornene-fused dibenzo[de,qr]tetracene nanobox
Source: Chem Sci. 2022 Jan 13;13(8):2280–5. doi: 10.1039/d1sc06553j (PMC8864699; doi:10.1039/d1sc06553j)
Supplement: SC-013-D1SC06553J-s001 [file SC-013-D1SC06553J-s001.pdf]

Electronic Supplementary Material (ESI) for Chemical Science.
This journal is © The Royal Society of Chemistry 2022

Supporting Information for

Synthesis, Aromatization and Cavitates of an Oxanorbornene-Fused Dibenzo[de, qr]tetracene Nanobox

Han Chen, Zeming Xia, Qian Miao*

Department of Chemistry, The Chinese University of Hong Kong
Shatin, New Territories, Hong Kong, China
* miaoqian@cuhk.edu.hk

Table of Contents

1. Synthesis 2
2. X-ray crystallography 5
3. Photophysical properties and fluorescence titration experiments 6
4. Density Function Theory (DFT) Calculations 12
5. High resolution MALDI-TOF mass spectra 14
6. NMR spectra 17
7. Reference 23

1. Synthesis

General: The reagents and starting materials employed were commercially available and used without any further purification or made following reported methods as indicated. Unless otherwise noted, all reactions were performed with dry solvents under an atmosphere of nitrogen in dried glassware with standard vacuum-line techniques. Anhydrous and oxygen-free CH_3CN, THF and CH_2Cl_2 were purified by an Innovative Technology Pure-Solv PS-MD-4 system. Gel permeation chromatography (GPC) were performed on a JAI LC-9160 II NEXT automatic recycling preparative HPLC system with a UV/VIS detector. NMR spectra were recorded on a Bruker AVANCE III 400MHz spectrometer (^{1}H NMR: 400 MHz, ^{13}C NMR: 100 MHz). Abbreviations: s = singlet, d = doublet, t = triplet, m = multiplet. Chemical shift values (δ) are expressed in parts per million using residual solvent protons (^{1}H NMR, δ_H = 5.32 for CD_2Cl_2, δ_H = 7.26 for $CDCl_3$, δ_H = 7.14 and 7.40 for *o*-$C_6D_4Cl_2$, ^{13}C NMR, δ_C = 77.16 for $CDCl_3$) as internal standard. Mass spectra were recorded on Thermo Q Exactive Focus Orbitrap Mass spectrometer or a Bruker Autoflex speed MALDI-TOF spectrometer. Unless otherwise noted, melting points, without correction, were measured using a Nikon Polarized Light Microscope ECLIPSE 50i POL equipped with an INTEC HCS302 heating stage.

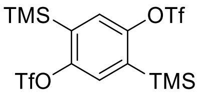

Trifluormethanesulfonic acid, 2,5-bis(trimethylsilyl)-1,4-phenylene ester (**5**) was synthesized following the reported procedures. [1]

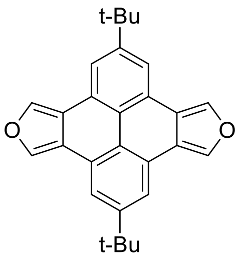

2,7-di-tert-butylpyreno[4,5-*c*:9,10-*c*']difuran (**6**) was synthesized following the reported procedures.[2]

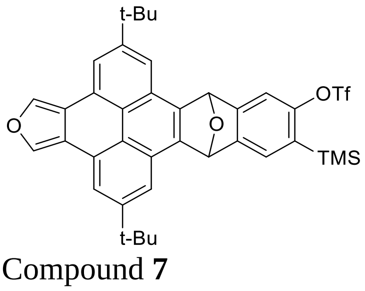

Compound **7**

A mixture of **5** (289mg, 0.55mmol), **6** (200 mg, 0.51 mmol) and caesium fluoride (92 mg, 0.6 mmol) was dissolved in a mixed solvent of acetonitrile (10 mL) and THF (2 mL), and the resulting mixture was stirred at room temperature overnight (about 12h). Then the reaction mixture was concentrated under a reduced pressure, and the crude product was purified by column chromatography on silica gel with hexane/CH_2Cl_2 2/1 (V/V) as eluent to afford 143 mg of **7** as white solid (41% based on **5** and 54% based on reacted **6**) together with 50 mg of **6** recovered. mp: decomposed upon heating to 250 °C. ^{1}H-NMR (400MHz, $CDCl_3$) δ (ppm): 8.42 (s, 2H), 8.25-8.24 (m, 2H), 8.03 (d, ^4J=1.6 Hz, 1H), 7.99 (d, ^4J=2 Hz, 1H), 7.49 (s, 1H),

7.42 (s, 1H), 6.78 (s, 1H),7.77 (s, 1H) 1.59 (s, 9H), 1.58 (s, 9H), 0.27 (s, 9H). ^{13}C-NMR (100MHz, $CDCl_3$) δ (ppm): 153.57, 152.57, 149.86, 149.72, 148.22, 145.93, 145.56, 136.94, 136.91, 129.19, 126.34, 126.00, 125.93, 125.86, 122.39, 122.28, 121.82, 119.58, 119.53, 117.37, 117.33, 112.64, 82.36, 82.15, 35.33, 31.86, 31.82, -0.76. HRMS(APCI): calcd. for $C_{38}H_{37}F_3O_5SSi$ ($[M+H]^+$): 691.2156, found: 691.2138.

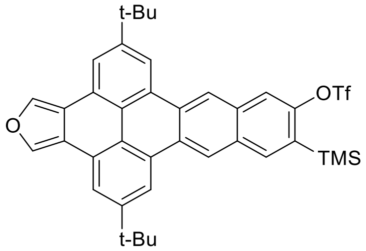

Compound **8**

To a mixture of **7** (300 mg, 0.43 mmol), $TiCp_2Cl_2$ (202 mg, 0.81 mmol) and activated Zn powder (130 mg, 2 mmol) was added 10 mL of anhydrous THF under an atmosphere of nitrogen, and the reaction mixture was stirred at 60 °C overnight. The resulting mixture was cooled to room temperature and diluted with CH_2Cl_2, and then filtered through a pad of celite to remove unreacted Zn powder. The filtrate was concentrated under a reduced pressure, and the residue was purified by flash chromatography on a short column of silica gel using hexane/CH_2Cl_2 2/1 (V/V) as eluent to give compound **8** (244mg, 83%) as pale yellow solid. mp: 280-284°C. ^{1}H-NMR($CDCl_3$) δ (ppm): 9.19 (s, 1H), 9.15(s, 1H), 8.86 (s, 2H), 8.43 (s, 2H), 8.33 (s, 1H), 8.24-8.23 (m, 2H), 8.13 (s, 1H), 1.64 (s, 18H), 0.53 (s, 9H). ^{13}C-NMR (100MHz, $CDCl_3$) δ (ppm): 152.8, 149.7, 149.6, 138.1, 136.6, 132.5, 131.4, 130.8, 130.4, 130.1, 129.4, 129.1, 125.5, 125.4, 122.7, 122.6, 122.4, 122.2, 121.7, 120.8, 120.6, 118.8, 118.5, 116.2, 35.45, 31.9, -0.45. HRMS(APCI): calcd. for $C_{38}H_{37}F_3O_4SSi$ ($[M+H]^+$): 675.2208, found: 675.2191.

Compound **3** and **9**

Compound **8** (50 mg, 0.074 mmol) and caesium fluoride (56 mg, 0.37 mmol) were dissolved in a mixed solvent of acetonitrile (20 mL) and THF (20 mL). The reaction mixture was stirred at 50°C for 4 days, and then concentrated under reduced pressure. The crude product was purified by column chromatography on silica gel with CH_2Cl_2 as eluent to afford **3** (2.8 mg, 8%) and **9** (3 mg, 9%) as pale yellow solids.

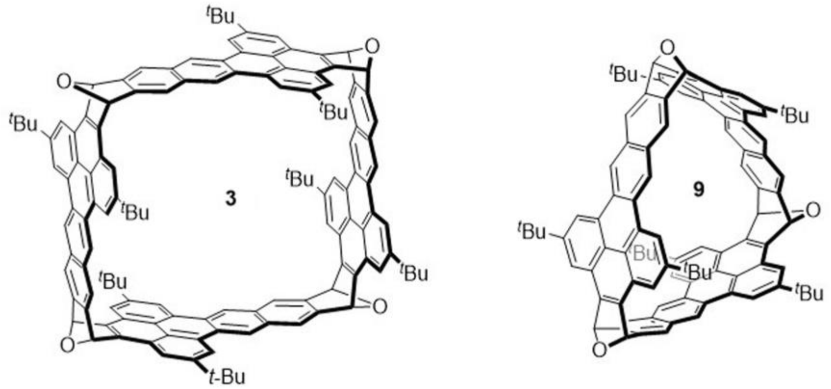

Compound **3** mp: not melt when heated up to 300 °C. ^{1}H-NMR($CDCl_3$) δ (ppm): 8.87(s, 8H), 8.81(s, 8H), 8.20(s,8H), 7.88(s, 8H), 6.97(s,8H), 1.62(s, 8H). Due to the poor solubility in $CDCl_3$, ^{13}C NMR spectrum with acceptable resolution was not obtained after 15000 scans due to the low solubility. HRMS (MALDI-TOF): calcd. for $C_{136}H_{112}O_4$ ($[M]^+$): 1809.8589, found: 1809.8590.

Compound **9** mp: not melt when heated up to 300 °C. ^{1}H-NMR($CDCl_3$) δ (ppm): 8.60(d, ^{4}J=1.2 Hz , 6H), 8.47(s, 6H), 8.10(d, ^{4}J=1.6 Hz, 6H), 7.54(s, 6H), 6.94(s, 6H), 1.57(s, 54H). ^{13}C-NMR (100MHz, $CDCl_3$) δ (ppm): 149.0, 144.7, 144.6, 131.1, 129.6, 129.1, 125.3, 123.4, 122.5, 118.6, 118.3, 117.9, 82.7, 35.5, 32.0. HRMS (MALDI-TOF): calcd. for $C_{102}H_{84}O_3$ ($[M]^+$): 1357.6449, found: 1357.6433.

Reductive aromatization of **3**

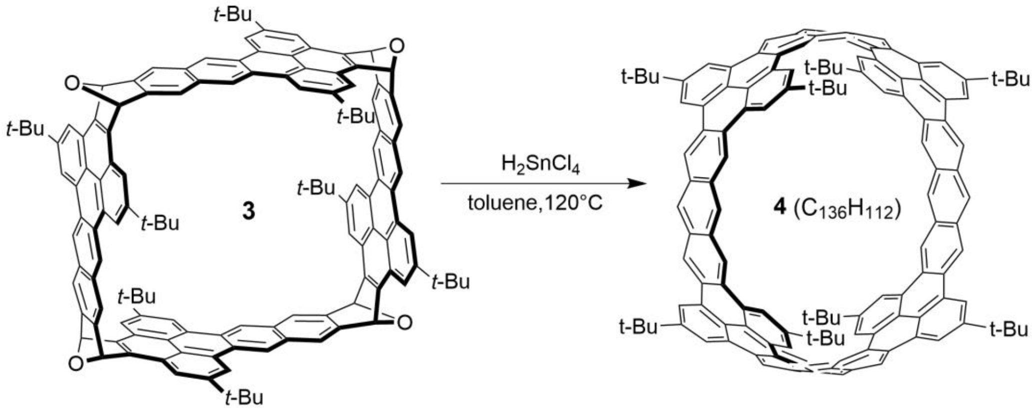

Solution of H_2SnCl_4 in THF was freshly prepared by dissolving anhydrous $SnCl_2$ (110 mg, 0.6 mmol) in 10 mL of anhydrous THF under an atmosphere of N_2 and adding 0.1 mL of concentrated aqueous hydrochloric acid to the solution. The resulting solution was stirred at room temperature for 20 minutes before use. Compound **3** (1.6 mg, 0.9 μmol) was dissolved in 3 mL of toluene under an atmosphere of N_2. To the resulting solution was added 0.1 mL of the freshly prepared solution of H_2SnCl_4 in THF (6 μmol of H_2SnCl_4). After being stirred at 120°C under the same atmosphere of N_2 for 18 hours, this solution changed from colorless to yellowish. The crude product was characterized with MALDI-TOF mass spectroscopy. HRMS (MALDI-TOF): calcd. for $C_{136}H_{112}$ ($[M]^+$): 1745.8792, found: 1745.8791.

2. X-ray crystallography

X-ray crystallography data were collected on a Bruker AXS Kappa ApexII Duo Diffractometer.

Table S1. Crystal data and structure refinement for **3** and **C_{60}⊂3**

compound	**3**·5($C_2H_4Cl_2$)·2(CH_2Cl_2)	C_{60}⊂**3**·2($C_2H_4Cl_2$)·2(C_7H_8)
Empirical formula	C_{136} $H_{112}O_4$·5($C_2H_4Cl_2$)·2(CH_2Cl_2)	$C_{136}H_{112}O_4$·C_{60}·2($C_2H_4Cl_2$)·2(C_7H_8)
Formula weight	2474.86	2913.02
Temperature	173(2) K	162(2) K
Wavelength	0.71073 Å	0.71073 Å
Crystal system	Triclinic	Monoclinic
Space group	P-1	P2/c
Unit Cell Lengths (Å)	a = 14.1531(19) Å	a = 16.9220(15) Å
	b = 17.009(3) Å	b = 17.1293(18) Å
	c = 17.275(3) Å	c = 27.104(3) Å
Unit Cell Angle (°)	a= 93.079(7)°.	a= 90°.
	b= 100.115(6)°.	b= 100.952(4)°.
	g = 110.482(6)°.	g = 90°.
Volume	3805.2(10) $Å^3$	7713.3(14) $Å^3$
Z	1	2
Density (calculated)	1.080 Mg/m^3	1.254 Mg/m^3
Absorption coefficient	0.300 mm^{-1}	0.140 mm^{-1}
F(000)	1294	3040
Crystal size	0.500 x 0.400 x 0.300 mm^3	0.400 x 0.300 x 0.200 mm^3
Theta range for data collection	2.368 to 25.250°.	2.378 to 25.250°.
Reflections collected	77111	118035
Independent reflections	13553 [R(int) = 0.1409]	13958 [R(int) = 0.1774]
Completeness to theta = 25.242°	98.50%	99.70%
Absorption correction	multi-scan	multi-scan
Max. and min. transmission	0.7456 and 0.5806	0.7456 and 0.6687
Refinement method	Full-matrix least-squares on F^2	Full-matrix least-squares on F^2
Data / restraints / parameters	13553 / 13 / 761	13958 / 94 / 1000
Goodness-of-fit on F2	1.36	1.05
Final R indices [I>2sigma(I)]	R1 = 0.1522, wR2 = 0.3929	R1 = 0.1808, wR2 = 0.4293
R indices (all data)	R1 = 0.2498, wR2 = 0.4503	R1 = 0.2620, wR2 = 0.4849
Extinction coefficient	n/a	n/a
Largest diff. peak and hole	0.775 and -0.846 e.$Å^{-3}$	0.929 and -0.829 e.$Å^{-3}$

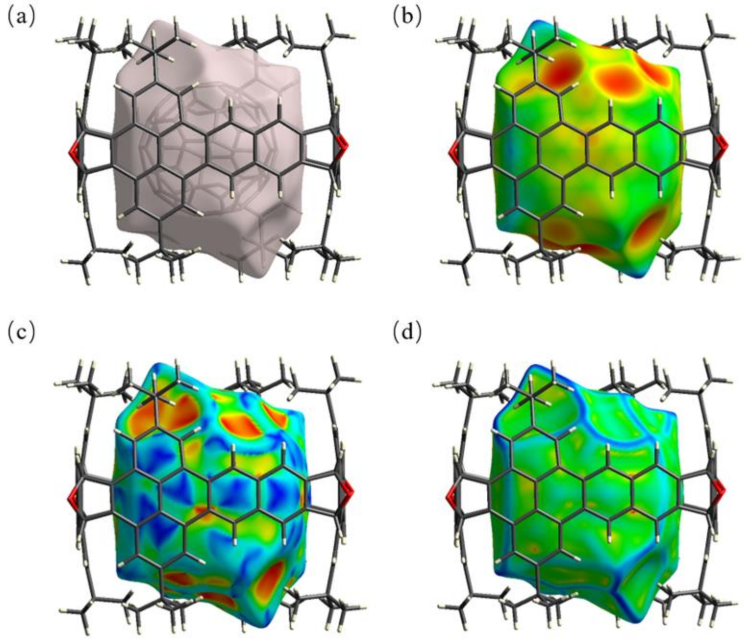

Figure S1. Crystallographic analysis by Hirshfeld surfaces of $\mathbf{C_{60}{\subset}3}$**.** Color mappings are shown for (b) d_e (minimum distance: 1.15 Å in red, maximum distance: 3.36 Å in blue), (c) shape index (minimum value: -1 in red, maximum value: 1 in blue) (d) and curvedness (minimum value: -3.4 in red, maximum value: 0.25 in blue).[3]

3. Photophysical properties and fluorescence titration experiments

UV-vis absorption spectra were recorded on a Shimadzu UV-3600 plus UV-VIS-NIR spectrophotometer. Fluorescence spectra were recorded on a Hitachi F-4500 spectrofluorometer.

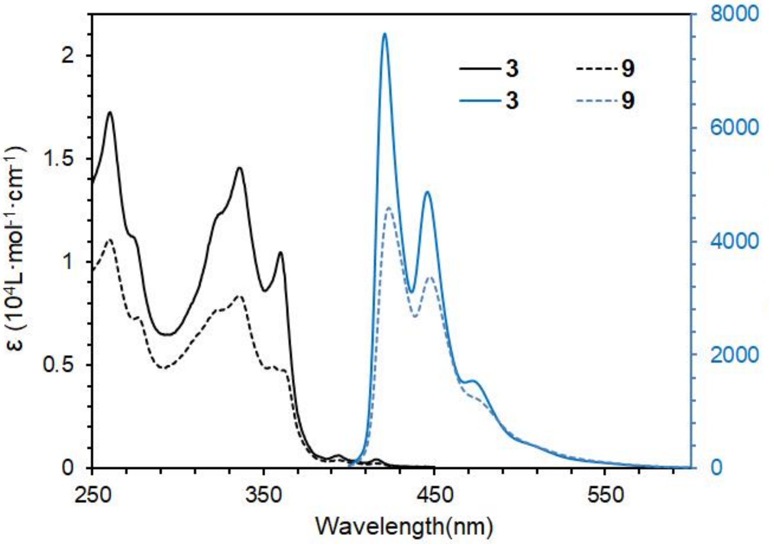

Figure S2. Absorption spectra and photoluminescence spectra of **3** (solid line) and **9** (dashed line) in CH_2Cl_2 (1×10^{-5} mol/L). (The photoluminescence spectra of **3** and **9** were measured with excitation at 360 nm)

Fluorescence titration experiments

Job's plot

A solution of nanobox **3** in toluene and another solution of fullerene (C_{60}, C_{70}, PCBM or ICBA) in toluene were mixed in different ratios to prepare 10 samples with a fixed total concentration (C_{60} and **3**: 4.4 × 10^{-6} mol/L; ICBA and **3**: 1.0 × 10^{-5} mol/L; PCBM and **3**: 1.0 × 10^{-5} mol/L; C_{70} and **3**: 1.0 × 10^{-7} mol/L). The fluorescence of each sample was measured, and the changes of fluorescence intensity (at 420 nm) were monitored for Job's plot analysis. Results indicate that each fullerene adopts a 1:1 binding stoichiometry with **3** in toluene.

Determination of binding constant

The binding constant was determined from multiple fluorescence titration experiments, in which a solution of host molecule (**3**) was titrated with a variable amount of fullerenes in toluene. Three independent titration experiments were conducted for the host, and the initial concentration of **3** included 5.0×10^{-7} mol/L (for C_{60}, ICBA and PCBM) and 1.0×10^{-7} mol/L (for **C_{70}**). The change in the fluorescence (*ΔF*) of the hosts (at 420 nm) was plotted against the concentration of fullerenes. On the basis of 1:1 complex model, association constant K_a is calculated by non-linear curve fitting using the following equation:

$$F/F_0=(1+(kf/k_s)K_a[L])/(1+K_a[L])$$

where F, F_0, *kf*, *ks*, $[L]$ and K_a are fluorescence intensity, fluorescence intensity of the nanobox before the addition of fullerenes, proportionality constant of the complex, proportionality constant of the host, the concentration of the guest, and the binding constant, respectively.[4]

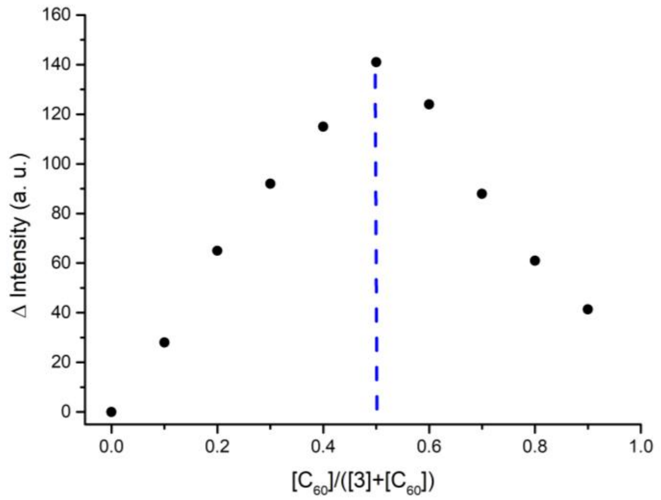

Figure S3. Job's plot based on the fluorescence change at 420 nm for **3** with C_{60}, indicating a 1:1 binding stoichiometry between **3** and C_{60} in toluene.

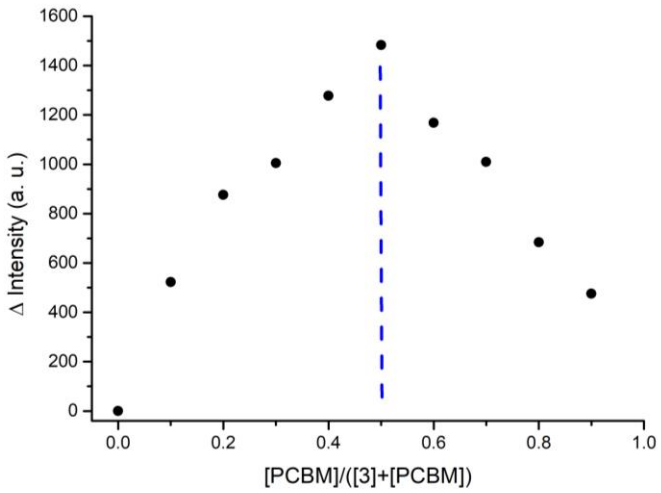

Figure S4. Job's plot based on the fluorescence change at 420 nm for **3** with PCBM, indicating a 1:1 binding stoichiometry between **3** and PCBM in toluene.

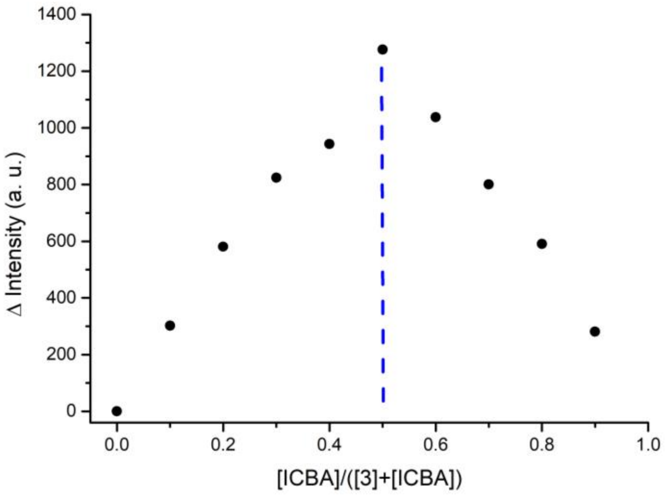

Figure S5. Job's plot based on the fluorescence change at 420 nm for **3** with ICBA, indicating a 1:1 binding stoichiometry between **3** and ICBA in toluene.

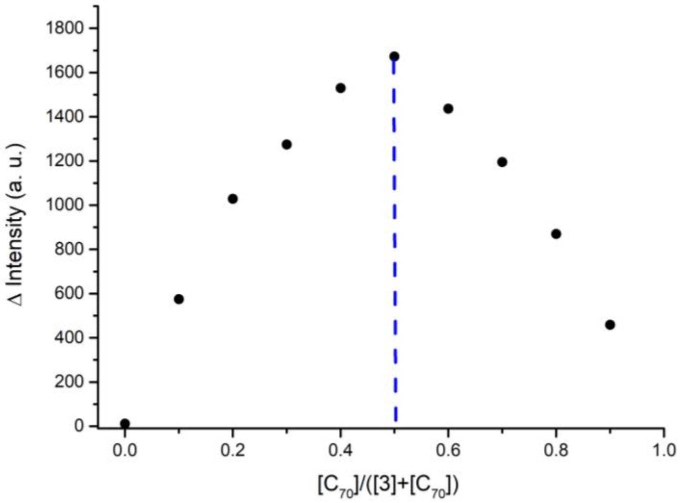

Figure S6. Job's plot based on the fluorescence change at 420 nm for **3** with C_{70}, indicating a 1:1 binding stoichiometry between **3** and C_{70} in toluene.

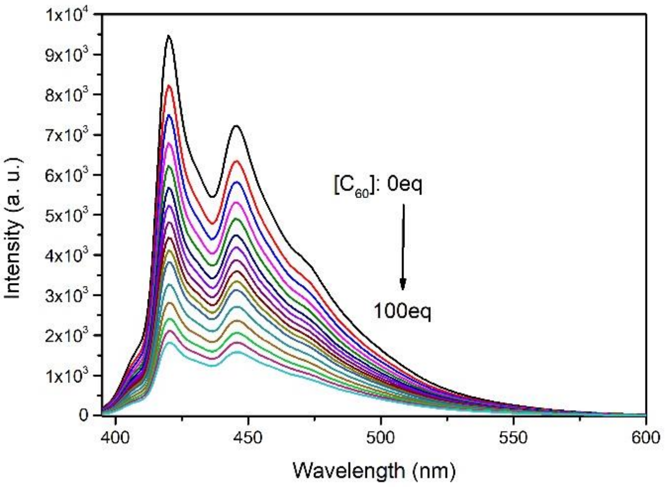

Figure S7. Fluorescence spectra of **3** (5.0×10^{-7} mol/L) in toluene in the presence of C_{60} (from 0 to 5.0×10^{-5} mol/L).

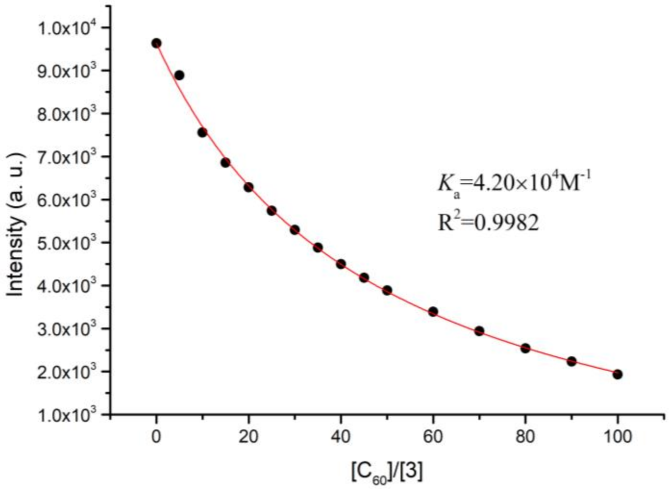

Figure S8. Nonlinear curve regression for a titration experiment of **3** with C_{60} using 1:1 binding model. Based on this data set, K_a is calculated to be 4.2×10^4 M^{-1}.

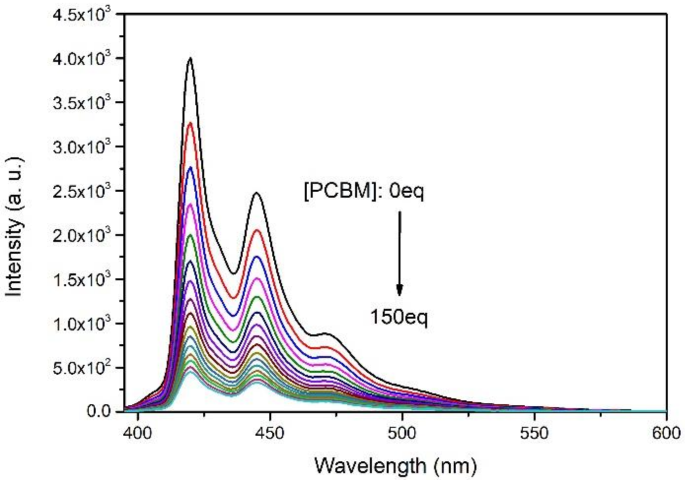

Figure S9. Fluorescence spectra of **3** (5.0×10^{-7} mol/L) in toluene in the presence of PCBM (from 0 to 7.5×10^{-5} mol/L).

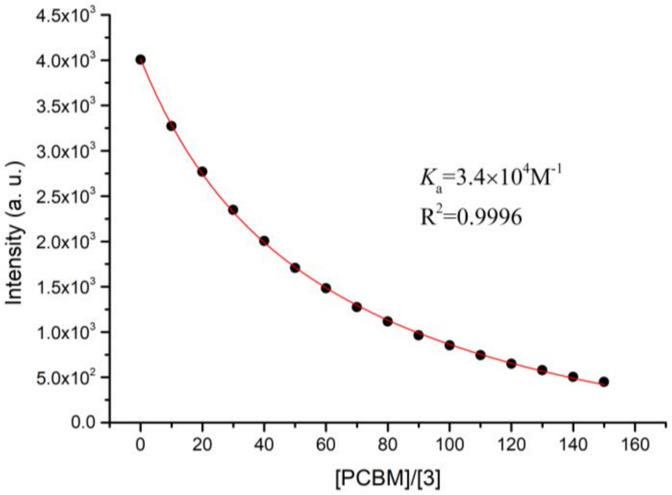

Figure S10. Nonlinear curve regression for a titration experiment of **3** with PCBM using 1:1 binding model. Based on this data set, K_a is calculated to be 3.4×10^4 M^{-1}.

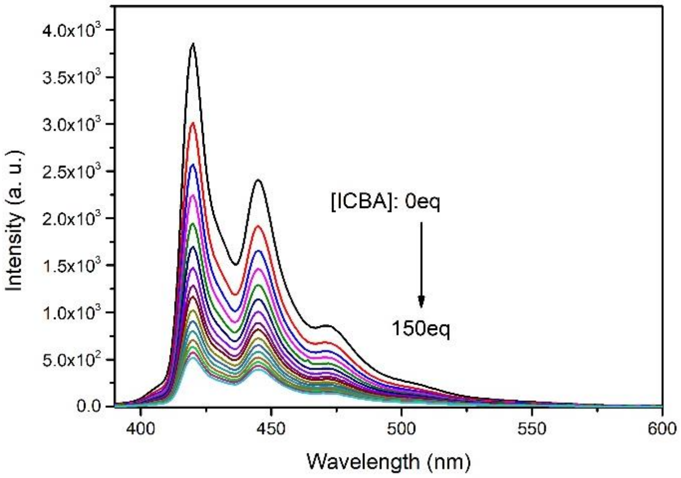

Figure S11. Fluorescence spectra of **3** (5.0×10^{-7} mol/L) in toluene in the presence of ICBA (from 0 to 7.5×10^{-5} mol/L).

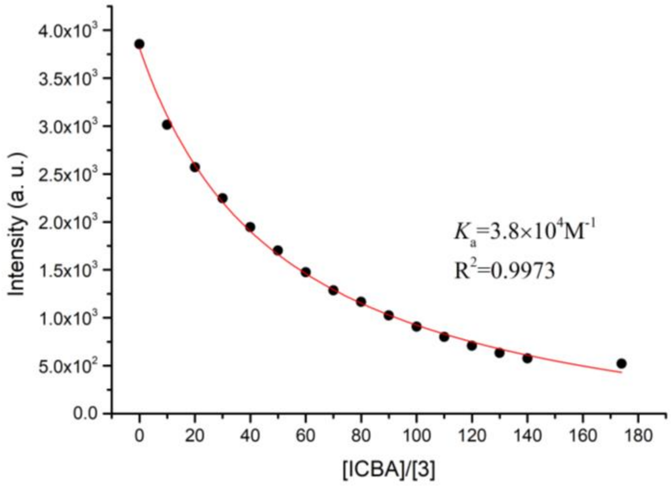

Figure S12. Nonlinear curve regression for a titration experiment of **3** with ICBA using 1:1 binding model. Based on this data set, K_a is calculated to be 3.8×10^4 M^{-1}.

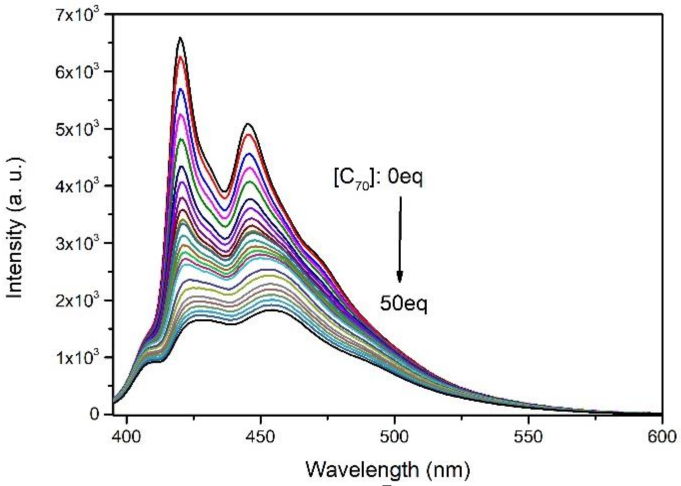

Figure S13. Fluorescence spectra of **3** (1.0×10^{-7} mol/L) in toluene in the presence of C_{70} (from 0 to 5.0×10^{-6} mol/L).

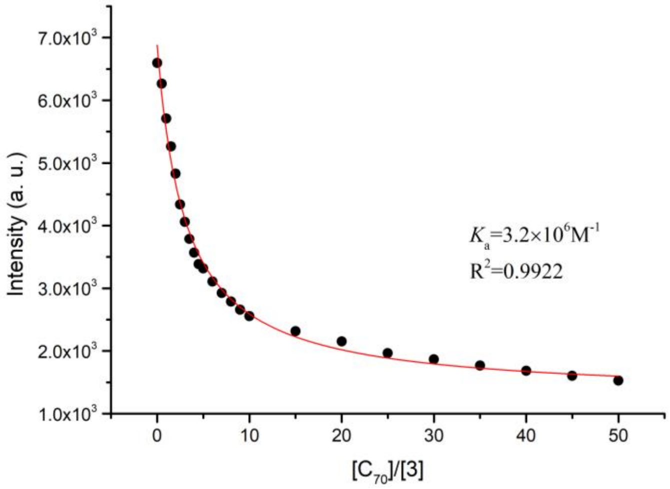

Figure S14. Nonlinear curve regression for a titration experiment of **3** with C_{70} using 1:1 binding model. Based on this data set, K_a is calculated to be 3.2×10^6 M^{-1}.

Table S2. Binding constants (K_a) and energy (ΔG) of carbon nanobox **3** for different fullerene derivtives

	K_a (M^{-1})	ΔG (kcal/mol)
**C_{60}⊂3**	$(3.3 \pm 0.8)\times10^4$	-6.2 ± 0.1
**PCBM⊂3**	$(3.3 \pm 0.9)\times10^4$	-6.1 ± 0.1
**ICBA⊂3**	$(3.1 \pm 0.7)\times10^4$	-6.1 ± 0.1
**C_{70}⊂3**	$(3.2 \pm 0.08)\times10^6$	-8.9 ± 0.01

4. Density Function Theory (DFT) Calculations

All the DFT calculations were done by using Gaussian 09 program.[5]

Calculations of molecular orbitals

The frontier molecular orbitals of **3** and **9** were calculated using simplified model molecules **3'** and **9'**, which have smaller methyl groups replacing *t*-butyl groups to reduce the computational cost. Energy-minimized models of **3'** and **9'** and their molecular orbitals were calculated at the B3LYP /6-31g(d) level of DFT.

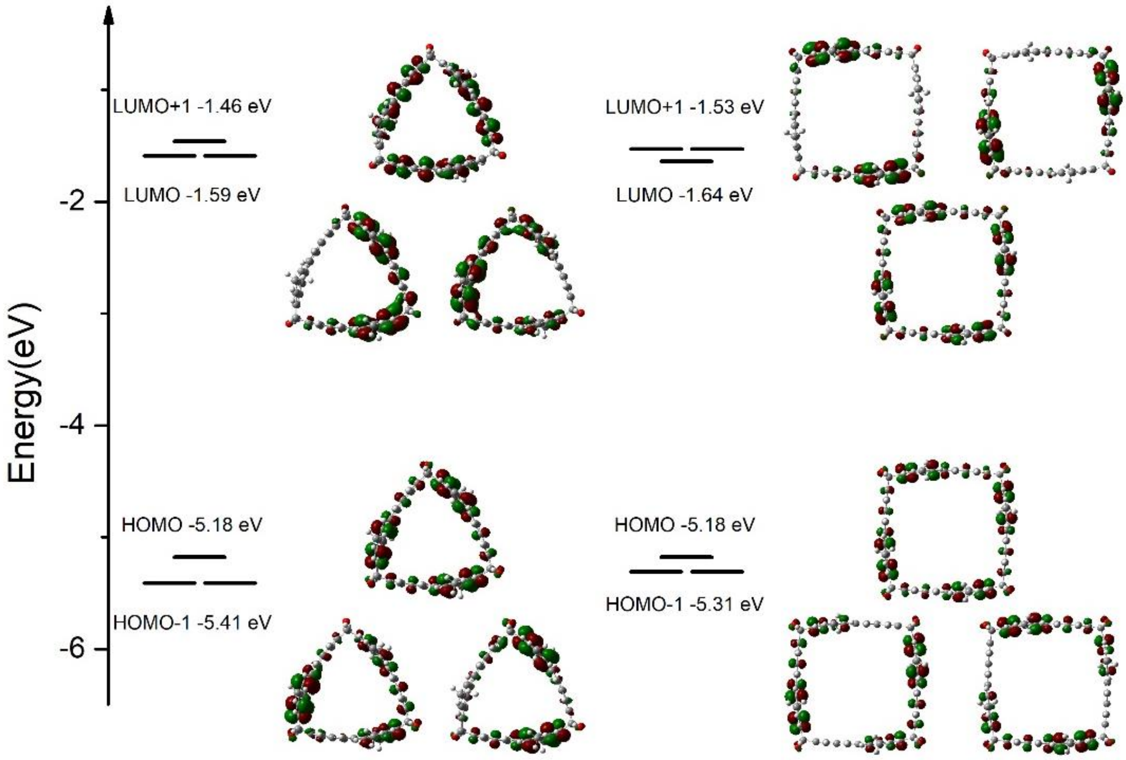

Figure S15. DFT calculations (B3LYP/6-31-g(d)) of molecular orbitals of **9'**(left) and **3'**(right)

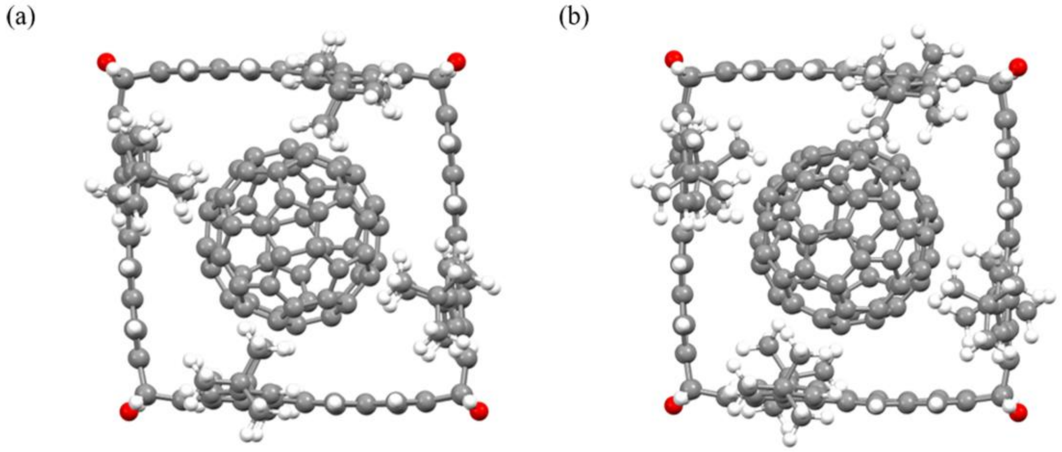

Figure S16. Calculated molecular model of (a) $\mathbf{C_{60}\subset 3}$ and (b) $\mathbf{C_{70}\subset 3}$ based on B3LYP function with empirical D3 dispersion correction (B3LYP-D3/6-31g(d)).

Strain energy

The strain energies of **3** and **9** were estimated on the basis of the following hypothetical homodesmotic reactions. The structures of the reactants and products of these reactions were calculated at the B3LYP level of DFT with the 6-31g(d) basis set, and were confirmed as true minima by vibrational analysis with no imaginary frequency. Based on the enthalpy changes of these hypothetical reactions, the strain energy was calculated as 7.0 kcal/mol for **3’’** and 16.0 kcal/mol for and **9’’**.

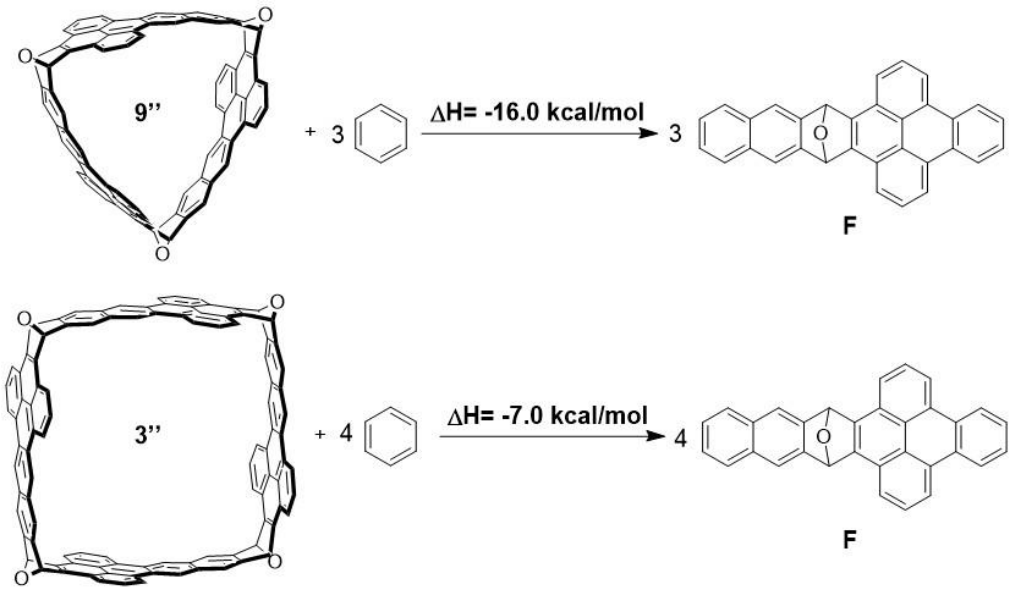

Figure S17. Hypothetical homodesmotic reactions with the enthalpy change for calculation for the strain energy of **9’’** and **3’’**.

5. High resolution MALDI-TOF mass spectra

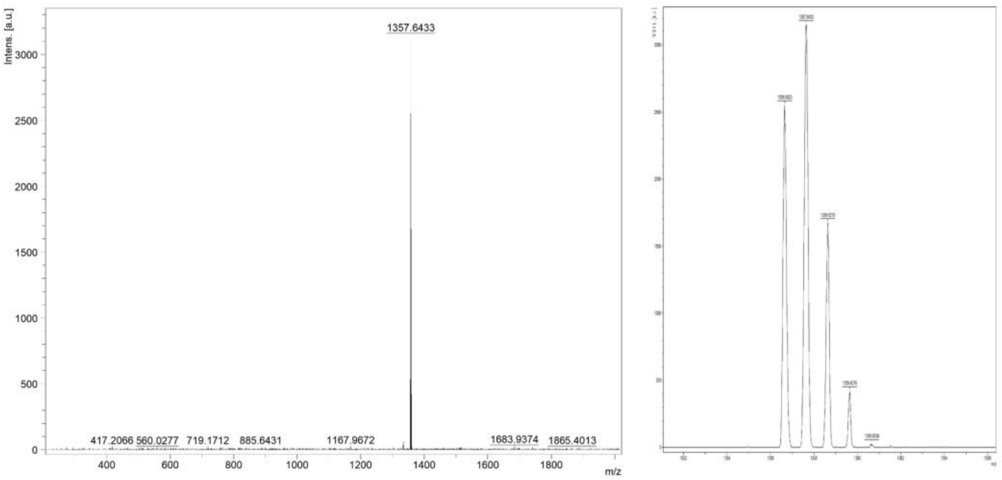

Figure S18. High resolution MALDI-TOF mass spectrum of **9** (calcd. for $C_{102}H_{84}O_3$ ($[M]^+$): 1357.6449, found: 1357.6433)

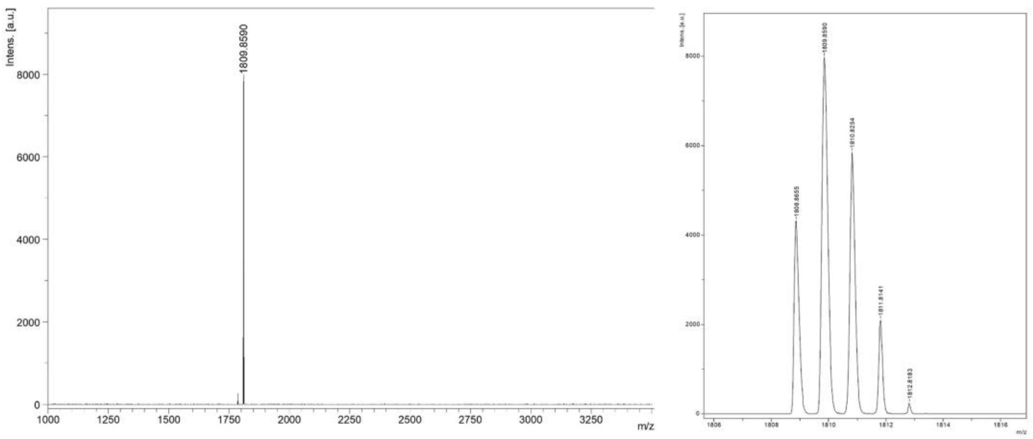

Figure S19. High resolution MALDI-TOF mass spectrum of **3** (calcd. for $C_{136}H_{112}O_4$ ($[M]^+$): 1809.8589, found: 1809.8590)

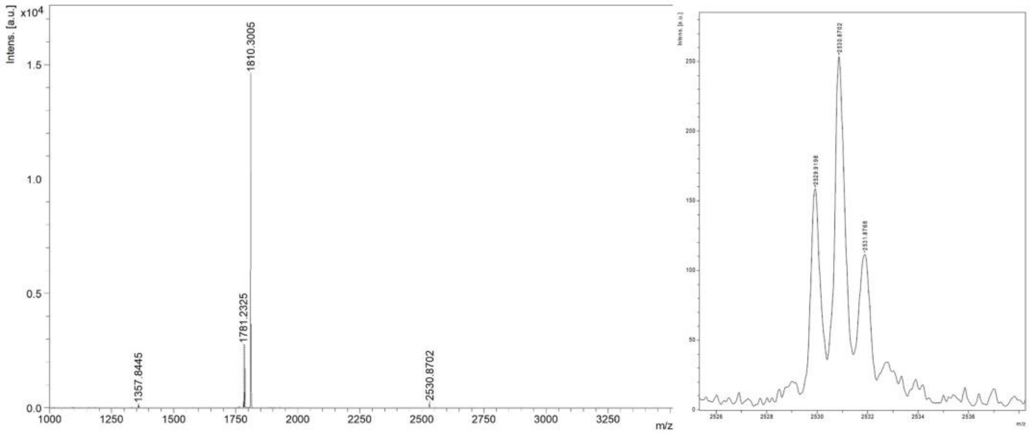

Figure S20. High resolution MALDI-TOF mass spectrum of **C$_{60}$⊂3** (calcd. for $C_{196}H_{112}O_4$ ($[M]^+$): 2530.8623, found: 2530.8702)

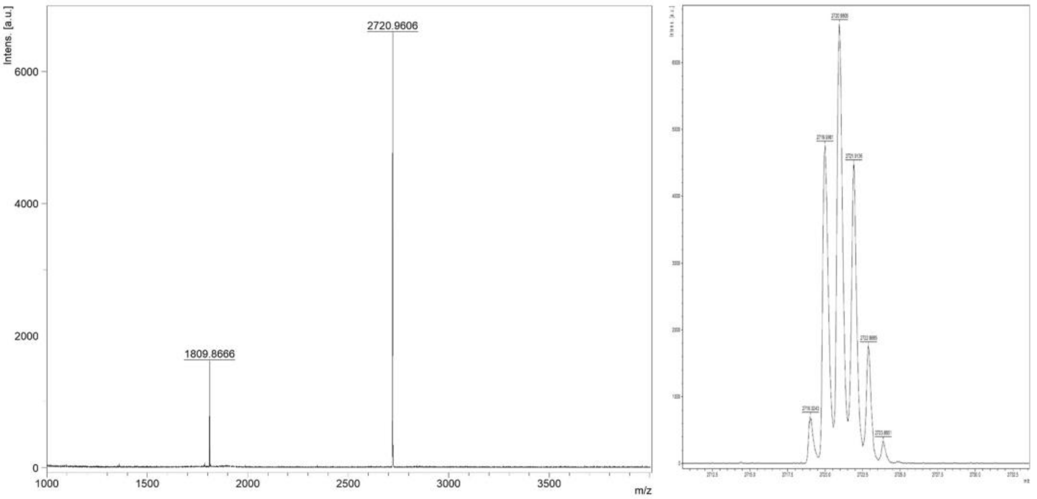

Figure S21. High resolution MALDI-TOF mass spectrum of **PCBM⊂3** (calcd. for $C_{208}H_{126}O_6$ ([M]$^+$): 2720.9616, found: 2720.9606)

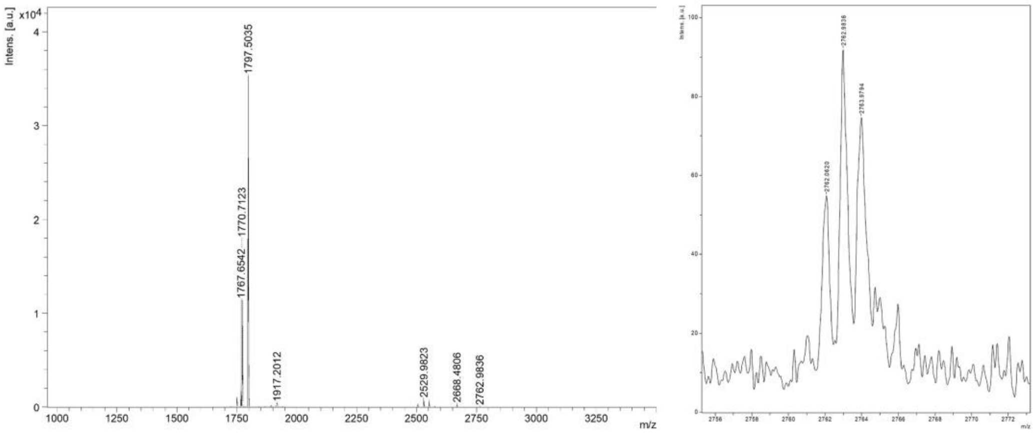

Figure S22. High resolution MALDI-TOF mass spectrum of **ICBA⊂3** (calcd. for $C_{214}H_{118}O_4$ ([M]$^+$): 2762.9875, found: 2762.9836)

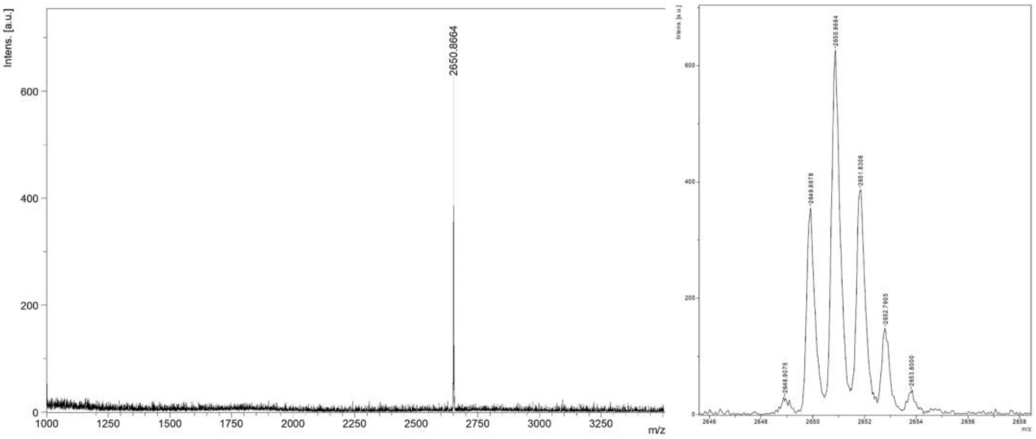

Figure S23. High resolution MALDI-TOF mass spectrum of **C$_{70}$⊂3** (calcd. for $C_{206}H_{112}O_4$ ([M]$^+$): 2650.8623, found: 2650.8664).

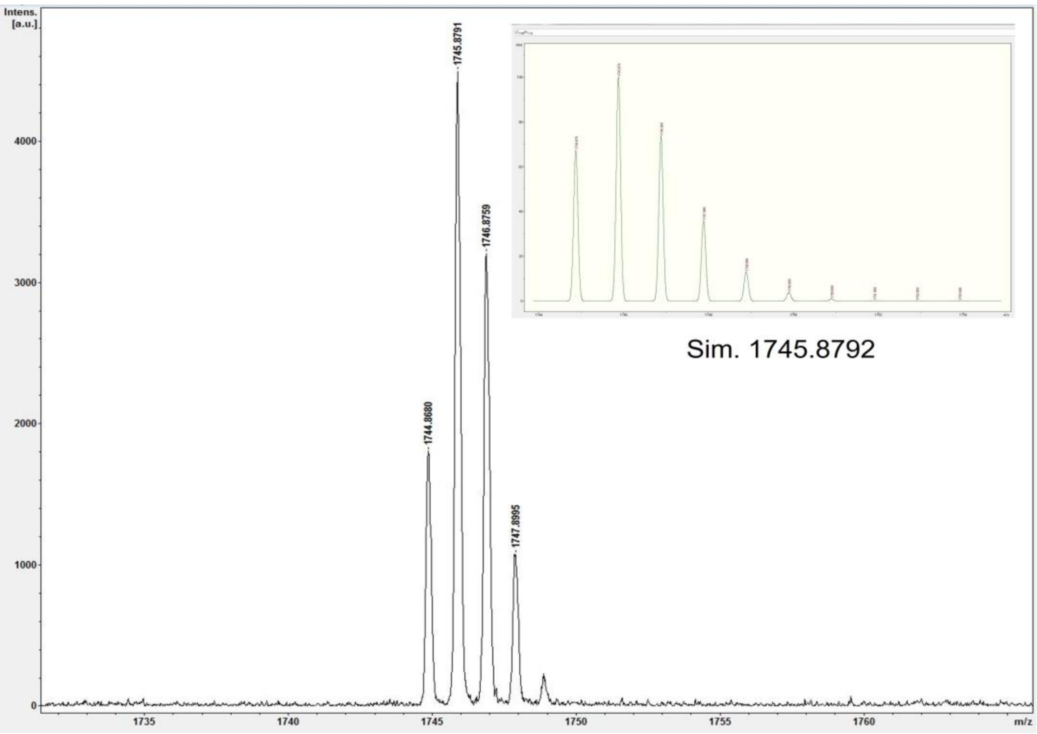

Figure S24. High resolution MALDI-TOF mass spectrum of the zigzag carbon nanobelt (calcd. for $C_{136}H_{112}$ ($[M]^+$): 1745.8792, found: 1745.8791)

6. NMR spectra

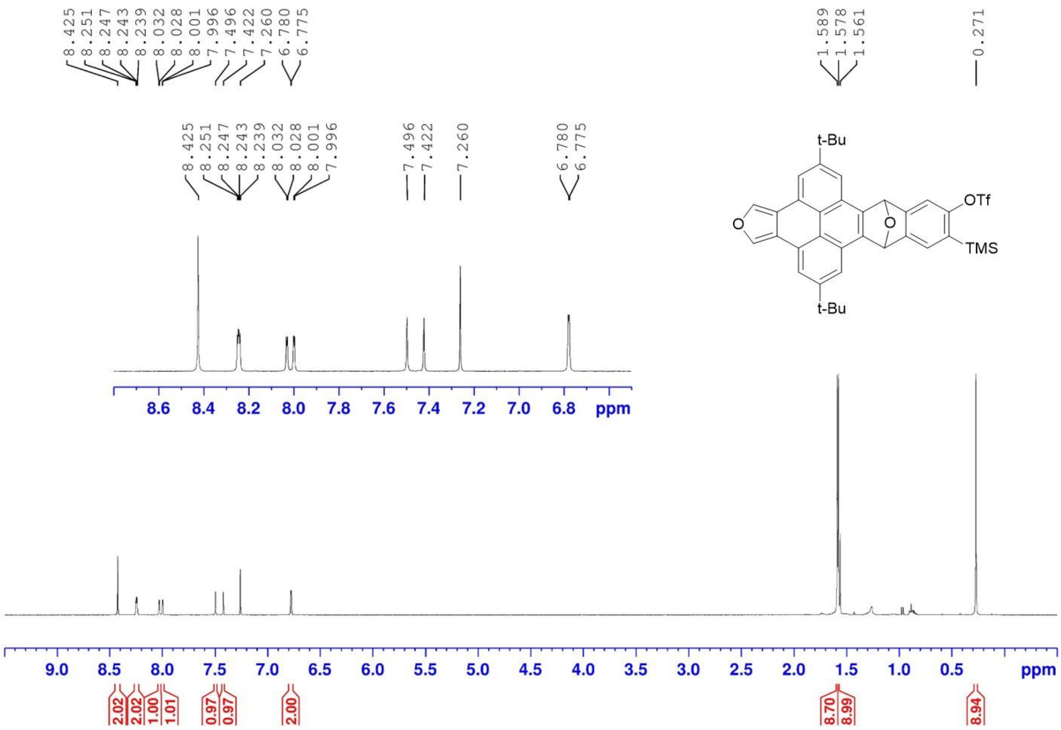

Figure S25. ^{1}H NMR spectrum (400MHz, $CDCl_3$) of **7**

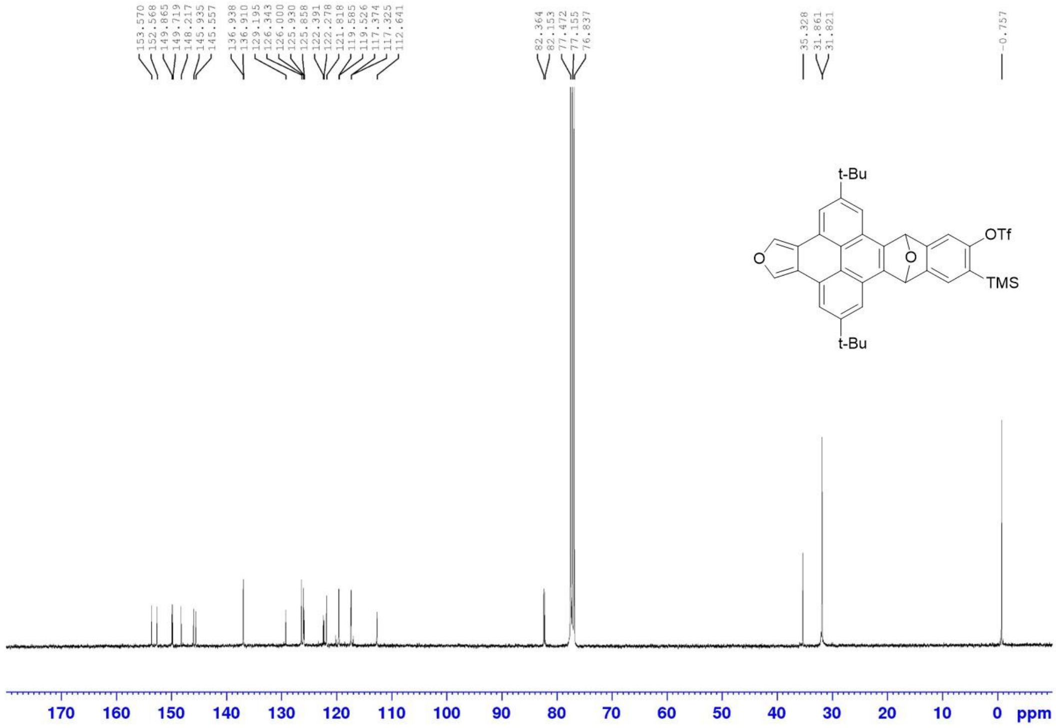

Figure S26. ^{13}C NMR spectrum (100MHz, $CDCl_3$) of **7**

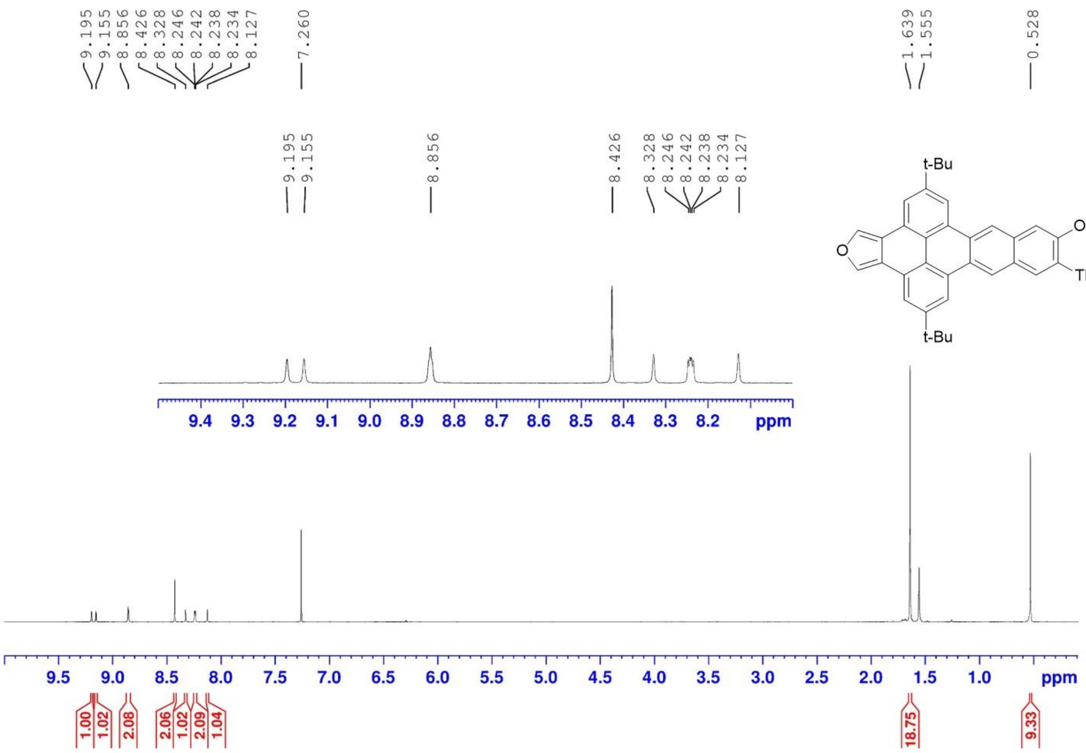

Figure S27. ^{1}H NMR spectrum (400MHz, $CDCl_3$) of **8**

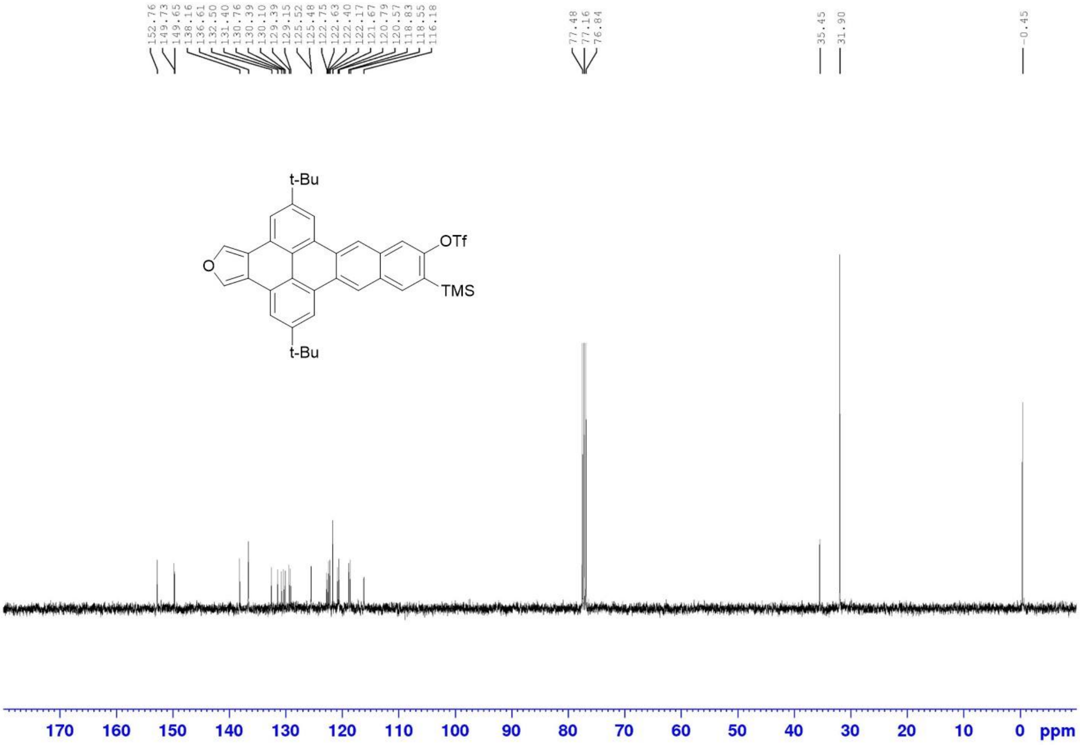

Figure S28. ^{13}C NMR spectrum (100MHz, $CDCl_3$) of **8**

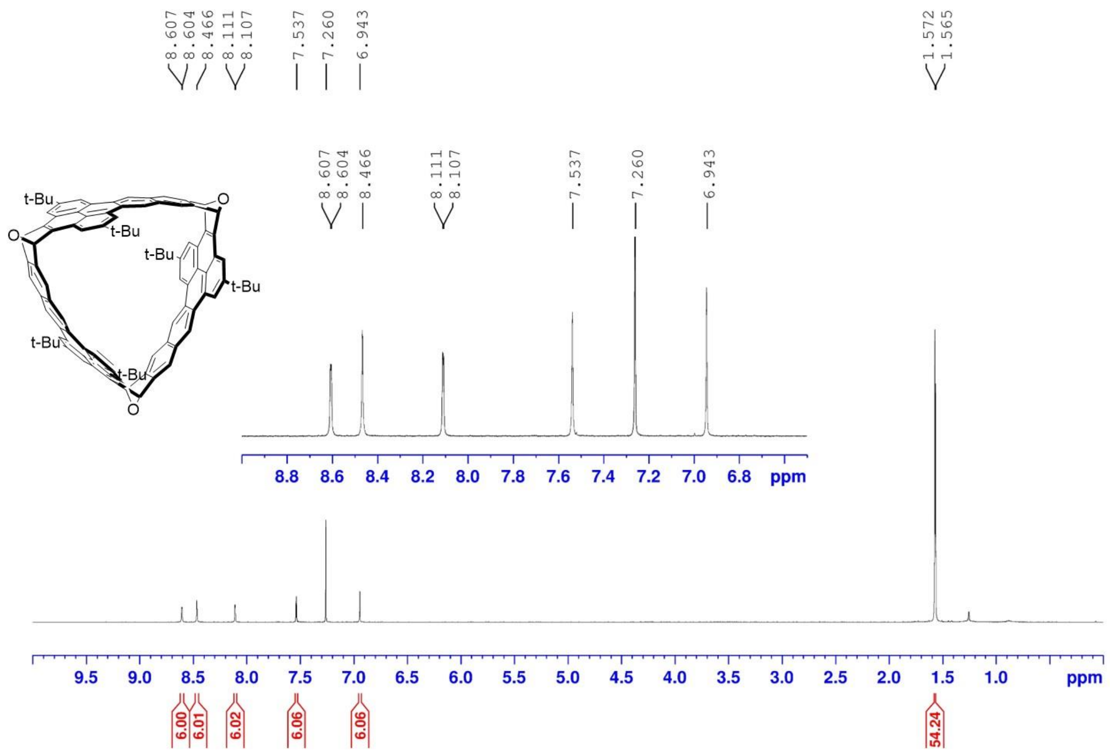

Figure S29. ^{1}H NMR spectrum (400MHz, $CDCl_3$) of **9**

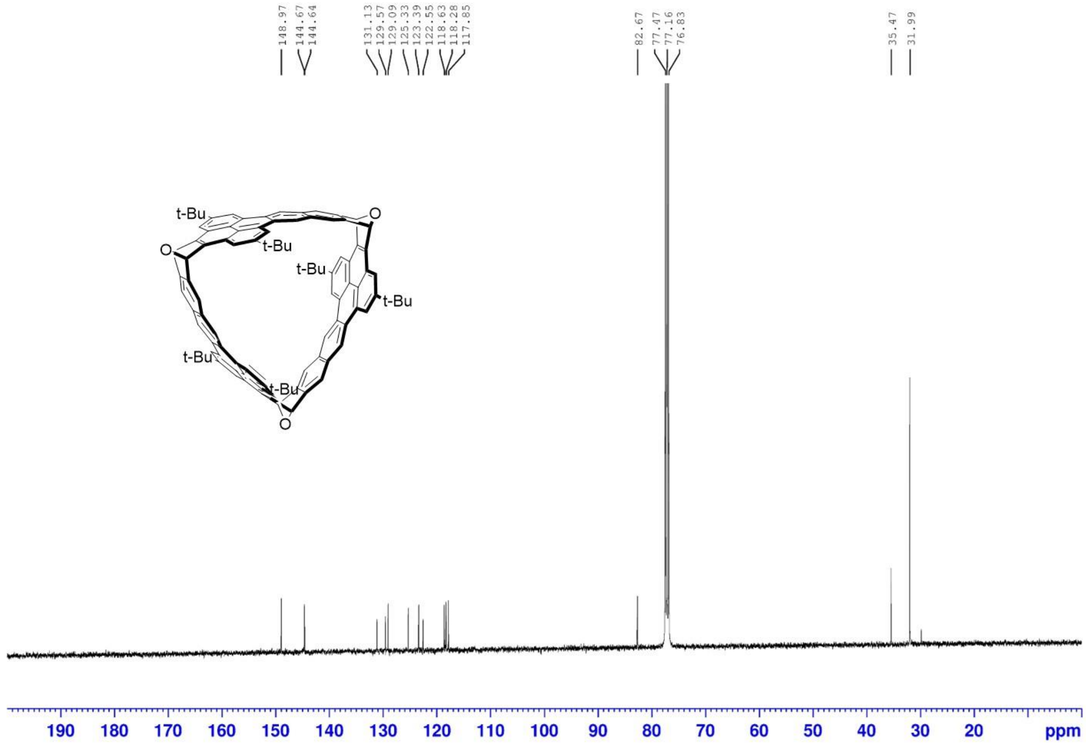

Figure S30. ^{13}C NMR spectrum (100MHz, $CDCl_3$) of **9**

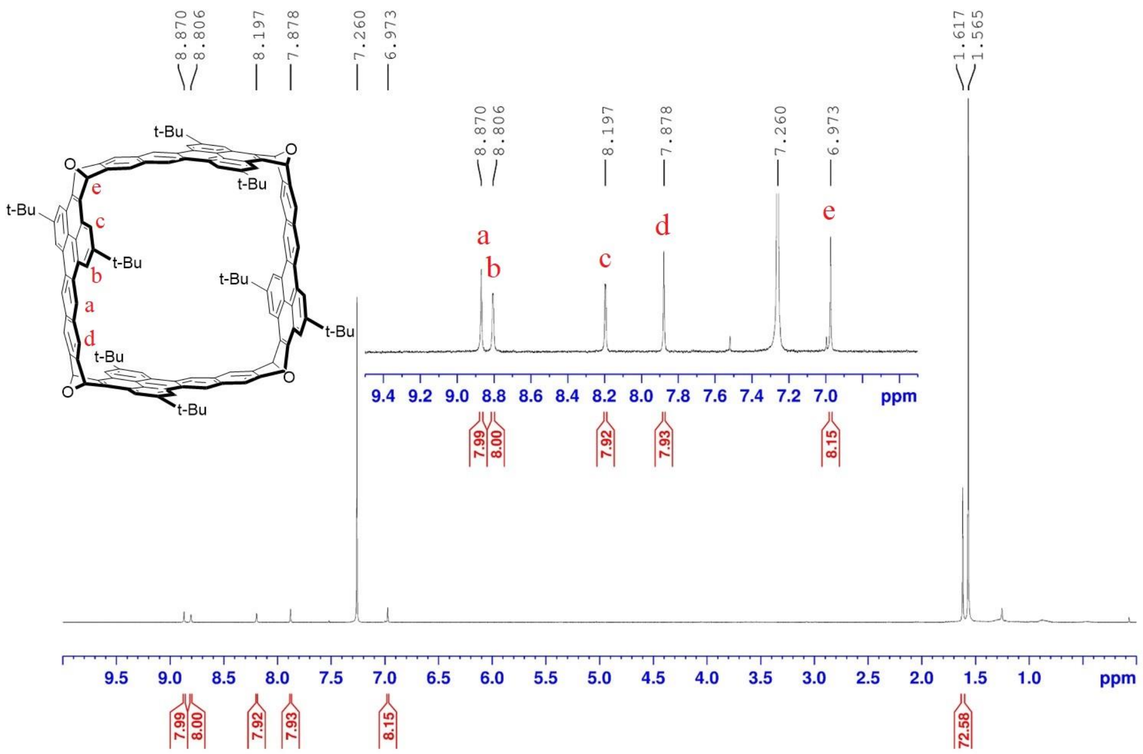

Figure S31. ^{1}H NMR spectrum (400MHz, $CDCl_3$) of **3**

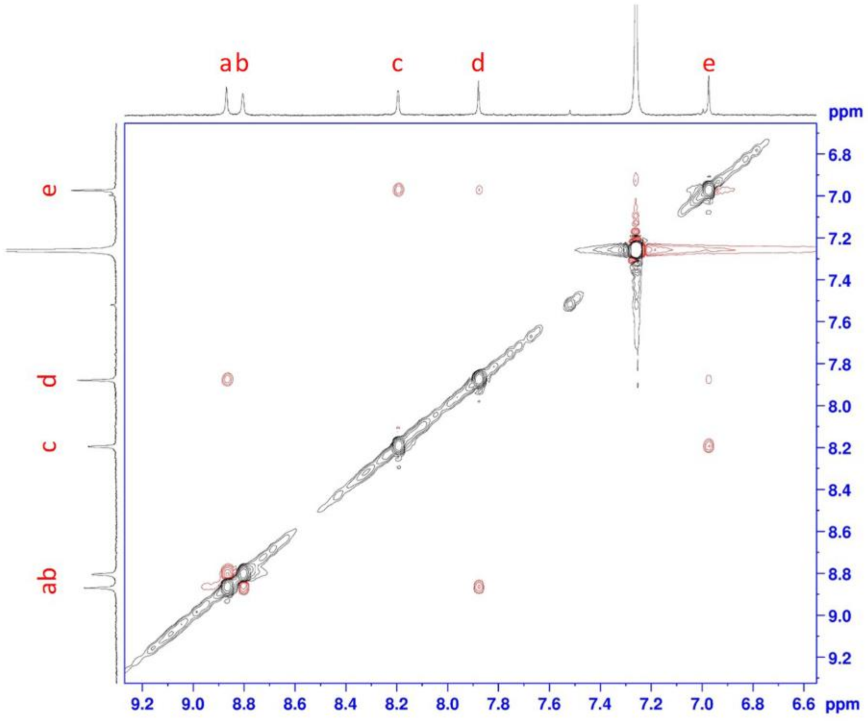

Figure S32. ROESY spectrum of **3** in CDCl3 at 298K (aromatic region)

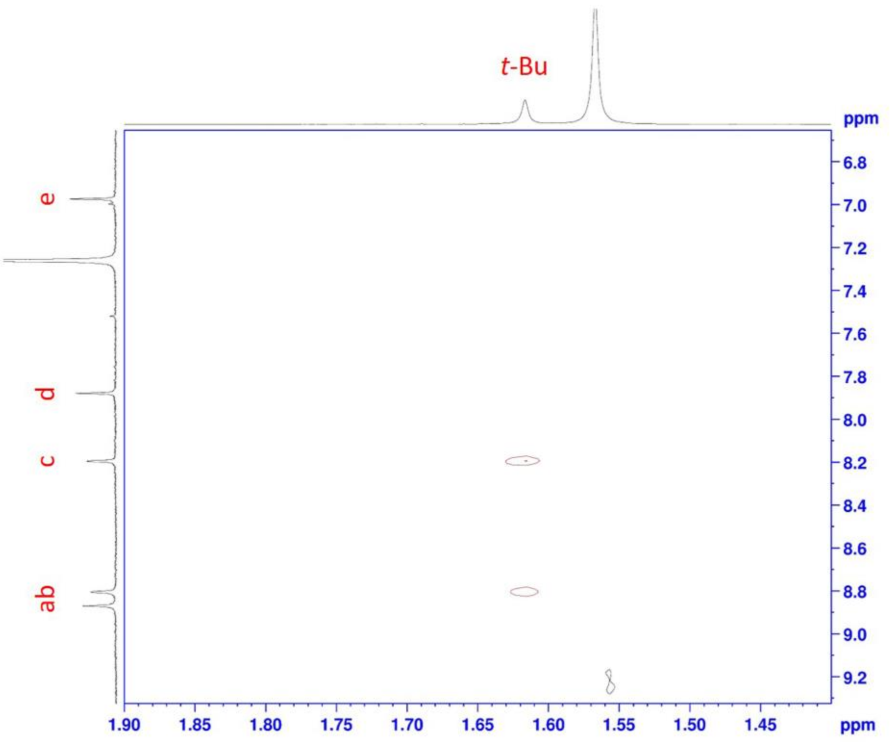

Figure S33. ROESY spectrum of **3** in CDCl3 at 298K (aromatic region versus aliphatic region)

NMR titration experiments

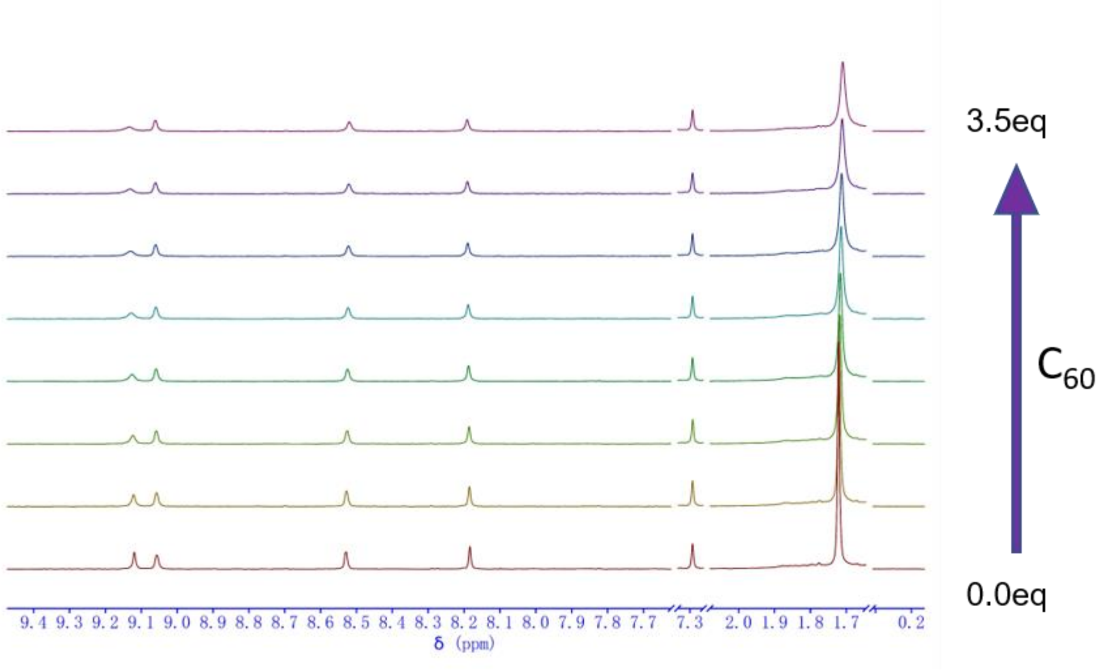

Figure S34. ^{1}H NMR spectrum (400MHz, $C_6D_4Cl_2$) of **3** as the increase of **C_{60}**

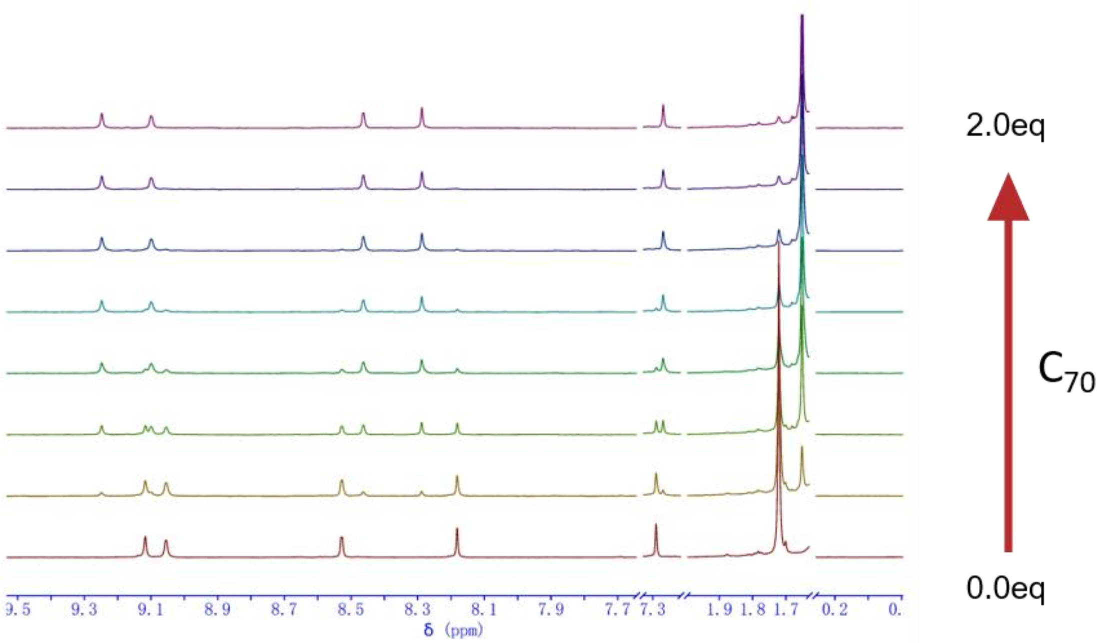

Figure S35. ^{1}H NMR spectrum (400MHz, $C_6D_4Cl_2$) of **3** as the increase of $\mathbf{C_{70}}$

The binding constant of C_{70}⊂**3** in *o*-dichlorobenzene was determined from NMR titration experiments, in which a solution of host molecule (**3**) (8.0×10^{-4} mol/L) was titrated with variable amounts of fullerenes (from 0 to 2 equivalents) in *o*-dichlorobenzene. On the basis of 1:1 complex model, association constant K_a is calculated by linear regression using the following equation:

$$[C] = -\frac{1}{K_a} \times \frac{[C]}{[H]} + [G_0]$$

where [C], [H], $[G_0]$ and K_a are the concentration of complex (C_{70}⊂**3**), host (**3**), initial concentration of guest (C_{70}) and the binding constant, respectively.

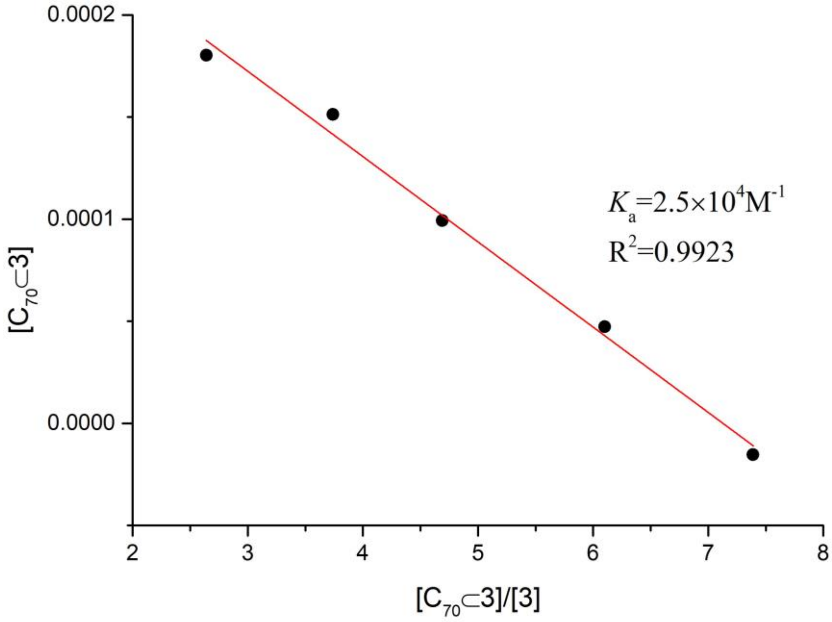

Figure S36. Linear curve regression for a titration experiment of **3** with C_{70} using 1:1 binding model. Based on the data set, K_a is calculated to be 2.5×10^4 M^{-1}.

7. Reference

(1) Pavliček, N.; Schuler, B.; Collazos, S.; Moll, N.; Pérez, D.; Guitián, E.; Meyer, G.; Peña, D.; Gross, L., On-surface generation and imaging of arynes by atomic force microscopy. *Nat. Chem.* **2015**, *7* (8), 623-628.

(2) Chen, H.; Gui, S.; Zhang, Y.; Liu, Z.; Miao, Q., Synthesis of a Hydrogenated Zigzag Carbon Nanobelt. *CCS Chem.* **2020**, *2*, 613-619.

(3) Wolff, S. K.; Grimwood, D. J.; McKinnon, J. J.; Turner, M. J.; Jayatilaka D. and Spackman, M. A., *CrystalExplorer*, *version 3.1.1*, University of Western Australia, **2012**.

(4) (a) Connors, K. A. *Binding Constants*; John Wiley and Sons: New York, 1987. (b) Iwamoto, T.; Watanabe, Y.; Sadahiro, T.; Haino, T.; Yamago, S., Size-selective encapsulation of C_{60} by [10]cycloparaphenylene: formation of the shortest fullerene-peapod. *Angew. Chem. Int. Ed.* **2011**, *50*, 8342-8344.

(5) Gaussian 09, Revision E.01, M. J. Frisch, G. W. Trucks, H. B. Schlegel, G. E. Scuseria, M. A. Robb, J. R. Cheeseman, G. Scalmani, V. Barone, G. A. Petersson, H. Nakatsuji, X. Li, M. Caricato, A. Marenich, J. Bloino, B. G. Janesko, R. Gomperts, B. Mennucci, H. P. Hratchian, J. V. Ortiz, A. F. Izmaylov, J. L. Sonnenberg, D. Williams-Young, F. Ding, F. Lipparini, F. Egidi, J. Goings, B. Peng, A. Petrone, T. Henderson, D. Ranasinghe, V. G. Zakrzewski, J. Gao, N. Rega, G. Zheng, W. Liang, M. Hada, M. Ehara, K. Toyota, R. Fukuda, J. Hasegawa, M. Ishida, T. Nakajima, Y. Honda, O. Kitao, H. Nakai, T. Vreven, K. Throssell, J. A. Montgomery, Jr., J. E. Peralta, F. Ogliaro, M. Bearpark, J. J. Heyd, E. Brothers, K. N. Kudin, V. N. Staroverov, T. Keith, R. Kobayashi, J. Normand, K. Raghavachari, A. Rendell, J. C. Burant, S. S. Iyengar, J. Tomasi, M. Cossi, J. M. Millam, M. Klene, C. Adamo, R. Cammi, J. W. Ochterski, R. L. Martin, K. Morokuma, O. Farkas, J. B. Foresman, and D. J. Fox, Gaussian, Inc., Wallingford CT, 2016.
